# Diversity of biofilm architectures within the genus *Tenacibaculum*

**DOI:** 10.1128/aem.01181-26

**Published:** 2026-07-31

**Authors:** Sabit Ahmed, Virgile Guéneau, Tatiana Rochat, Benjamin Fradet, Eric Leclercq, Eric Duchaud, Romain Briandet

**Affiliations:** 1 Université Paris-Saclay, INRAE, AgroParisTech, Micalis Institute, Jouy-en-Josas, France; 2 LabCom Biofilm1Health, Lallemand SAS & Micalis Institute, INRAE, AgroParisTech, Université Paris-Saclay 483681 , Jouy-en-Josas, France; 3 Lallemand SAS 483681 , Blagnac, France; 4 Université Paris-Saclay, INRAE, UVSQ, VIM, Jouy-en-Josas, France; Indiana University Bloomington, Bloomington, Indiana, USA

**Keywords:** CLSM, EPS, biofilms, *Tenacibaculum*, tenacibaculosis, aquaculture

## Abstract

Marine aquaculture sustainability is increasingly threatened by tenacibaculosis, a disease caused by *Tenacibaculum* species that are thought to persist through biofilm formation. However, the architectural diversity of biofilms across the genus and its contribution to persistence remain unclear. Here, we conducted a large-scale phenotypic analysis of 40 *Tenacibaculum* isolates including 19 *T. maritimum* strains, an aquaculture-relevant fish pathogen, and 21 type strains representing different species. Using three complementary growth models: planktonic free-cell cultures, macro-colony biofilms, and submerged biofilms, 17 quantitative parameters were measured. We observed substantial inter- and intra-species heterogeneity. Strains adopted distinct developmental phenotypes, ranging from a high-yield, slow-growth profile associated with thick, structured three-dimensional biofilms to fast-growing communities with limited architectural complexity. Confocal imaging revealed high structural variation across the genus, with Congo red-labeled extracellular materials exhibiting strain-specific differences in porosity and spatial distribution within the biofilm. Multivariate analysis partitioned the collection into four phenotypic clusters defined by distinct growth and structural signatures. Antimicrobial susceptibility assays using oxytetracycline hydrochloride in four representative strains showed that biofilm-associated tolerance was strain-dependent. Together, this work identifies biofilm architecture as a measurable and informative dimension of phenotypic diversification in *Tenacibaculum* and provides candidate structural markers to support future mechanistic studies and the development of strategies aimed at limiting the persistence of problematic *Tenacibaculum* species in aquaculture systems.

**IMPORTANCE:**

The genus *Tenacibaculum* includes several fish-pathogenic species that pose major concerns in marine aquaculture, yet their biofilm biology remains poorly characterized. By analyzing 40 isolates across complementary growth models, we show that biofilm architecture represents a quantifiable source of phenotypic variation within the genus. Strains adopted various developmental profiles that uncouple the growth rate, biomass yield, and three-dimensional structural complexity. Importantly, experiments performed on four representative strains indicate that biofilm-associated antimicrobial survival is strain-dependent and may be influenced by biofilm structural organization. These findings emphasize the need to account for structure-based functional profiling in addition to species-based classification. By identifying candidate architectural markers and representative phenotypic clusters, this work provides a rational framework for future mechanistic studies and for evaluating whether specific biofilm traits are associated with persistence or risk in aquaculture systems.

## INTRODUCTION

Aquaculture has emerged as a crucial food production sector that provides a significant portion of the net animal protein worldwide, hence making a major contribution to global food supply and dietary needs. It accounts for approximately 17% of the global animal protein intake and 7% of all protein consumed by humans, with over 3.3 billion people directly relying on fish for a significant portion of their dietary protein (1). According to the Food and Agriculture Organization (FAO), global fish production, which includes both wild-caught and farmed production, reached 175 million metric tons (Mt) in 2019 and is expected to rise by almost 15% by 2032 (1).

As aquaculture systems expand and intensify, they often result in demanding management conditions such as high rearing density, which can cause mechanical damage to the skin and mucus barrier of farmed fish (2, 3). In addition, the intensification of global trade has increased the movement of fish pathogens across borders, which has significantly increased infectious disease outbreaks in aquatic systems. Global warming has also led to environmental changes, such as rising seawater temperatures and degraded habitat conditions, which pose further stress to fish populations and create favorable conditions for the proliferation of pathogens (4). Altogether, these factors compromise the fish’s ability to maintain good health and markedly increase their susceptibility to infectious diseases. As a result, they pose a serious threat to the sustainability and productivity of aquaculture activities.

In this context, a major cause of poor animal welfare and economic losses in marine aquaculture is tenacibaculosis, a disease caused by pathogenic species of the genus *Tenacibaculum* belonging to the family *Flavobacteriaceae* (5). Clinical symptoms include ulcerative or necrotic skin lesions, ragged fins, an eroded or hemorrhagic mouth, and tail rot (6). While 38 validly named species of *Tenacibaculum* have been described to date, only a subset including *T. maritimum*, *T. discolor*, *T. soleae*, *T. gallaicum*, *T. finnmarkense*, *T. ovolyticum*, *T. dicentrarchi*, and *T. piscium* (5) are known fish pathogens.

Morphologically, *Tenacibaculum* cells are rod-shaped (0.1–0.7 μm in diameter and 0.4–40.0 μm in length), non-flagellated but motile by gliding, and occasionally pleomorphic (5, 7). Under environmental stress or starvation, *T. maritimum* has been observed to undergo a drastic transition from filamentous rods to spherical forms, potentially acting as a survival strategy to withstand adverse conditions (8). Despite lacking pili or flagella, gliding motility enables bacterial colonization through the use of outer membrane, mobile adhesins propelled by the type 9 secretion system (T9SS) (9). The iridescent phenotype observed in certain *Tenacibaculum* species is attributed to a dense hexagonal arrangement of bacterial cells that generates the structural colors (5).

Although pathogenic *Tenacibaculum* species are recognized as the primary agents of tenacibaculosis, their life strategies for survival and transmission remain largely unexplored. Current knowledge indicates that fish surfaces serve as primary infection sites (10) and that *T. maritimum,* one of the pathogenic species in the *Tenacibaculum* genus*,* can be isolated from sediments, tank surfaces, and water samples that have been in contact with infected fish stocks (4). Marine organisms and jellyfish blooms may also act as reservoirs and vectors (11).

Most available studies focus on *T. maritimum* (5), a filamentous, highly proteolytic pathogen measuring 2–30 µm in length and 0.5 µm in diameter (12, 13). Like other *Tenacibaculum* species, its genome encodes components of the T9SS and several T9SS cargo proteins with a C-terminal domain that serves as signals for secretion and cell-surface anchoring, including adhesins required for gliding motility (5, 14).

*T. maritimum* is highly aggregative, with strains forming dense cell clumps and a prominent ring of adherent cells at the interface between the broth and the culture flask wall (14). These microbial structures are biofilms. Such biofilm formation is a fundamental survival strategy in the microbial world. In the natural environment, microorganisms are predominantly members of spatially organized, matrix-enclosed communities known as biofilms (15, 16). These ubiquitous communities are essential to a wide range of natural processes, including the proper functioning of host-associated microbiota as well as the cycling of nutrients in ecosystems (15, 17). The proximity of cells in these biofilms leads to emergent properties and density-dependent communication mechanisms, such as quorum sensing, to ensure coordinated behaviors between various species (16).

In the specific context of *T. maritimum* and aquaculture, this transition to a biofilm lifestyle poses a dual challenge as it not only facilitates persistence in the environment, but it can also confer recalcitrance to antimicrobial treatments, making infected fish significantly harder to treat (18). Given that *T. maritimum* has been shown to survive poorly as free-living cells in non-sterile seawater, often remaining culturable for only a few days due to antagonism by native microbiota (8), biofilm formation likely provides a crucial survival advantage. This hypothesis is further supported by genomic studies identifying multiple genes associated with adhesins and polysaccharide biosynthesis (14), suggesting that *T. maritimum* is genetically primed to proliferate in an aggregated state as a preferred lifestyle and survival strategy against environmental stressors.

From another perspective, as *T. maritimum* forms biofilms, it produces extracellular polymeric substances (EPS), which may contribute to the local concentration of secreted virulence factors. For instance, binding to carbohydrate residues in skin mucus may be the first step in *T. maritimum* infection, and its progress will depend on the pathogen’s ability to evade immunological strategies and antimicrobial compounds in the mucus (18). It is already known that *T. maritimum* toxins play a key role during disease progression by further exacerbating bacterial colonization and invasion or by disintegrating and altering host tissues (19). This notion was further confirmed by Van Gelderen et al. (20) as they demonstrated that *T. maritimum* toxins indeed induce severe gill necrosis, cellular damage in the heart and pyloric ceca, as well as autolysis and rapid lysis of *Salmo salar* cells (20). Together, these findings strongly support the hypothesis that surface attachment and biofilm formation are central to the pathogenic lifestyle of *Tenacibaculum* species, experimentally demonstrated *via* the rapid biofilm development observed in *T. dicentrarchi* (21), and such biofilm-mediated adherence likely provides a specialized niche for environmental persistence and host colonization, ultimately facilitating further bacterial pathogenesis.

Although *Tenacibaculum* spp. have been well studied over the past decade, their biofilm-forming properties remain poorly understood. Current scientific evidence indicates that the dynamics of biofilm formation in *T. maritimum* create temporary reservoirs of pathogenic cells, ultimately intensifying tenacibaculosis outbreaks during the cell detachment phase (22). However, the in-depth mechanisms underlying this phenomenon remain unexplored. Therefore, further research into the biofilm life cycle of *T. maritimum* and other *Tenacibaculum* species is essential for elucidating their role in pathogenesis.

The present study explores the structural diversity of biofilms across the genus *Tenacibaculum*, including both clinical and environmental isolates, to address the critical knowledge gap on this important life state and assess its relationship with the ecological niche. Specifically, we assessed whether growth profiles and biofilm architectural diversification across strains are associated with responses to oxytetracycline-HCl (OTC-HCl). Oxytetracycline was selected because tetracycline-class antibiotics remain among the antimicrobial treatments historically and practically relevant for bacterial diseases in aquaculture (23), including tenacibaculosis-associated outbreaks. A comprehensive collection of 40 *Tenacibaculum* strains was carefully selected to include 22 clinical and 18 environmental isolates. This deliberate inclusion of diverse clinical and environmental strains, particularly the 19 *T. maritimum* isolates, is especially significant because this species demonstrates substantial intraspecific variability at both the genomic and serological levels (24, 25), with multiple recombining genetic subgroups and distinct O-antigen types that do not strictly correlate with host species, geography, or core-genome relatedness, therefore highlighting the necessity of including diverse isolates to adequately represent the variability and support meaningful comparative analyses of virulence traits. By characterizing the phenotypic diversity of *Tenacibaculum* spp. biofilms and integrating metadata with species-specific traits, this study aimed at revealing the links between biofilm formation, persistence, and pathogenesis, ultimately supporting the development of targeted control strategies for *Tenacibaculum*-related infections, thereby improving fish health and aquaculture sustainability.

Together, this work provides a quantitative phenotypic framework for describing biofilm diversity within the genus, identifying biomarkers associated with biofilms, and selecting strains for future mechanistic studies on persistence, matrix composition, and antimicrobial tolerance.

## MATERIALS AND METHODS

### Bacterial strains and culture conditions

The bacterial collection included isolates from marine environments and diseased fish, comprising 19 *T. maritimum* strains and 21 strains from other *Tenacibaculum* species (Table S1). Strains were stored at −80°C in medium supplemented with glycerol 20%. Strains were streaked on Marine Agar (BD Difco) and incubated at 28°C for 48 h. This temperature was selected to approximate conditions commonly encountered in temperate marine aquaculture systems. For liquid cultures, three colonies were inoculated into 7 mL of Marine Broth (BD Difco) previously sterilized by autoclaving, followed by filtration through a 0.22-µm filter in order to remove undissolved particles. All cultures were incubated at 28°C for 48 h with agitation at 150 rpm. These cultures were used as inocula for subsequent experiments.

### Phylogenetic analysis

The whole-genome sequences of the 40 *Tenacibaculum* strains were retrieved from the databases listed in Table S1. Genomic relationships among *Tenacibaculum* strains were assessed using the Mash software (26), which estimates pairwise genomic distances based on k-mer similarity. A neighbor-joining phylogenetic tree was constructed from the Mash distance matrix using the JavaScript package V 1.0.4 (https://github.com/biosustain/neighbor-joining). Branch length reflects 1–ANI (average nucleotide identity).

### Planktonic growth assay

The 48-h liquid cultures were diluted in filter-sterilized marine broth to a starting OD_600_ of 0.001. Aliquots (200 µL) were distributed into 96-well flat-bottom sterile microplates (Greiner Bio-One, France). Growth was monitored using a microplate reader set to 28°C with continuous double-orbital shaking. OD_600_ readings were acquired automatically every 20 min over 72 h. Each strain was assessed using three biological replicates (independent cultures), each with three technical replicates.

### Macro-colony biofilm formation

To study surface-associated biofilm formation and phenotypic heterogeneity across *Tenacibaculum* strains, a macro-colony biofilm model was used. Macro-colonies grown on solid surfaces recapitulate key biofilm features including structural complexity and matrix-dependent morphogenesis (27–30). Macro-colony biofilms were cultivated on marine agar using the Reshape Biotech imaging platform (Reshape, Denmark). To prepare the agar plates, stock marine agar bottles were kept in a hot water bath at 80°C for 2 h to allow sedimentation of undissolved marine salts. The clear supernatant was decanted and used to fill 12-well plates (Sarstedt, flat-bottom) with 2 mL agar per well. This clarification step minimized salt particle interference during automated image analysis.

The 3-µL aliquots of 48-h liquid cultures were spotted onto the center of each agar well. Plates were incubated at 28°C (static) within the Reshape Biotech automated imaging system, which captured images hourly for 7 days (Video S1). At least five biological replicates were prepared per strain.

Colony areas were automatically annotated and measured in real time using a built-in deep learning algorithm integrated into the Reshape pipeline (Fig. S1). To characterize the visual color profile of macro-colonies, a custom Python script (see Data availability, below) was utilized to manually delineate colony boundaries and extract mean HEX color values from top- and bottom-illuminated images acquired with the Reshape Biotech imaging platform.

### Growth curve analysis: planktonic and macro-colony models

Growth curves from both planktonic and macro-colony experiments were fitted to the Baranyi and Roberts primary growth model (31):


(1) $$\;\frac{1}{N(t)}\frac{dN(t)}{dt}=\mu_{\max}\cdot \alpha (t)\cdot f(t)$$

The model defines μ_max_ as the exponential growth rate, α(*t*) as the adjustment function, and *f*(*t*) as the inhibition function. This model provides estimates for μ_max_, lag phase [derived from α(*t*)], maximum population size [N_max_ derived from *f*(*t*)], and initial population size (N_0_). This established mathematical framework allowed for the extraction of key physiological parameters. For planktonic growth, the maximum growth rate (μ_max_) and the maximum optical density (N_max_) were precisely determined from the resulting growth curves.

For macro-colony expansion, the colony area over time was used to derive the apparent maximum growth rate (apparent μ_max_) and apparent maximum colony size (apparent N_max_) analogs. All model-fitting procedures were performed using custom Python scripts (see Data availability, below).

### Submerged biofilm formation assays

Submerged biofilms were cultivated at the bottom of μClear 96-well plates (Greiner Bio-One, France), compatible with high-resolution fluorescence microscopy. Liquid cultures (48 h) were diluted in filter-sterilized marine broth to achieve an OD_600_ of 0.001, and 200 µL of these suspensions was added to each well. Plates were incubated statically at 28°C for 1.5 h to allow cell adhesion. Afterward, the supernatant was carefully removed to eliminate non-adherent cells, and 200 µL of fresh sterile marine broth was added. Plates were incubated statically at 28°C, and biofilms were analyzed at T0 (immediately post-adhesion), T24 (24 h post-adhesion), and T72 (72 h post-adhesion). Three biological replicates were prepared per strain and time point, with two technical replicates per replicate.

### Confocal laser scanning microscopy (CLSM)

Prior to imaging, biofilms were stained with SYTO9 (Invitrogen, USA), a cell-permeant green dye that binds to nucleic acid, Congo red (Sigma-Aldrich, Germany) to contrast proteinaceous and polysaccharidic extracellular matrix components (32), or TOTO-3 (Invitrogen, USA) to visualize extracellular DNA (eDNA) (33). Fifty microliters of the staining solution containing 4 µg/mL SYTO9, 40 µg/mL Congo red, or 4 µg/mL TOTO-3 was added directly to each well.

Fluorescent imaging was conducted using a Leica SP8 AOBS inverted high-content screening confocal laser scanning microscope (HCS-CLSM, LEICA Microsystems, Germany) at the INRAE MIMA2 platform (https://doi.org/10.15454/1.5572348210007727E12). Images were acquired using a 63× water-immersion objective (NA 1.2) at 600 Hz, with z-stacks captured at 1-µm intervals. Each image had a resolution of 512 × 512 pixels (field of view: 184.52 µm × 184.52 µm; pixel size: 0.36 µm). SYTO9 and Congo red were excited with a 488-nm argon laser and TOTO-3 with a 633-nm HeNe laser. Emitted fluorescence was collected in the range 500–550 nm for SYTO9, 600–750 nm for Congo red and TOTO-3 using hybrid detectors (HyD LEICA Microsystems, Germany). Each condition was imaged across three biological replicates, with four technical replicates per biological replicate (12 z-stacks per condition).

### CLSM image analysis

Quantitative analysis of the acquired CLSM image stacks was performed using BiofilmQ software (v1.0.1) (34). Following this analysis, IMARIS 9.3.1 software (Bitplane, Zurich, Switzerland) was utilized for 3D reconstruction and visualization of biofilm structure, as well as for generating 2D projections from the image stacks (35). The analysis workflow in BiofilmQ involved the separate analysis of each fluorescence channel, corresponding to the different stains used. The image stacks were binarized using the OTSU thresholding algorithm, and the “global biofilm properties” function within BiofilmQ was used to extract descriptive parameters (35, 36). Key biofilm structural parameters were quantified, including total biovolume (µm³/µm²), mean thickness (µm^2^), maximum height (µm), surface area coverage (expressed as a unitless arbitrary unit, a.u.), and surface roughness (a.u.).

### Comparative antibiotic susceptibility of planktonic and biofilm cultures

The MIC of oxytetracycline-HCl (OTC-HCl) was determined against *T. mesophilum* CIP 107215T (TMP), *T. maritimum* 190605A03b (TM16), *T. crassostreae* JO1T (TCR), and *T. xiamenense* LMG 27422T (TX). Two-fold serial dilutions of the antibiotic were prepared in filter-sterilized marine broth. Assays were performed in sterile 96-well flat-bottom microplates (Greiner Bio-One, France) by mixing 100 μL of antibiotic solution with 100 μL of the inoculum prepared from 2-day cultures grown in filter-sterilized marine broth at 28°C with shaking and standardized to OD₆₀₀ =0.05. The final well volume was 200 μL, with strain-specific antibiotic gradients ranging from 0.25 to 256 μg/mL. For growth controls, strains were grown in antibiotic-free marine broth. Plates were incubated at 28°C with double orbital shaking, and the optical density at 600 nm was recorded automatically every 20 min for 72 h. Based on EUCAST guidelines (https://www.eucast.org/), the MIC was determined as the lowest concentration of OTC-HCl resulting in ≥80% growth inhibition relative to the antibiotic-free growth control. The assay was performed with at least four independent biological replicates per strain.

To evaluate the impact of biofilm lifestyle on antibiotic tolerance, planktonic and established biofilm cultures of TMP, TM16, TCR, and TX were challenged with OTC-HCl at their respective consensus MICs.

For planktonic form, bacterial suspensions were standardized to an OD_600_ of 0.05 prior to inoculation by dilution of a preculture grown in filter-sterilized marine broth for 2 days at 28°C with shaking. For planktonic assays, 200 µL of the culture was incubated in 1.5-mL Eppendorf tubes for 24 h at 28°C with continuous shaking at 150 rpm. Following incubation, cells were harvested by centrifugation, and the resulting pellets were resuspended in 200 µL of either antibiotic-supplemented or antibiotic-free filter-sterilized marine broth. The cultures were incubated for a further 24 h at 28°C. To quantify their viability, cells were harvested by centrifugation, the supernatant was discarded, and the pellets were resuspended in 200 μL of sterile physiological water. Biofilms were generated in microtiter plates using the previously described protocol. After 24 h of growth, the supernatants were removed and replaced with 200 μL of either filter-sterilized marine broth or medium containing OTC-HCl at the consensus MIC. The biofilms were incubated for 24 h at 28°C. After incubation, the supernatant was removed, and biofilms were resuspended in 200 µL of sterile physiological water by mechanical disruption. This was achieved by scraping the bottom of each well (area = 0.32 cm^2^) with a pipette tip (10 vertical and 10 horizontal strokes) while performing successive aspiration-dispensing cycles. Survivors were enumerated at the end of the experiment on marine agar by serial dilution and spot plating. The log reduction was calculated relative to the respective antibiotic-free growth controls. All assays were performed with three independent biological replicates and two technical replicates per condition.

### Pan-genome analysis

The whole-genome sequences of the 40 *Tenacibaculum* strains listed in Table S1 were used for genome annotation, pan-genome comparisons, and extraction of core-genome, variable-genome, and strain-specific coding DNA sequence (CDS) using the MicroScope platform (37) following an established pipeline described by reference 38. A cutoff of 80% identity and 80% on the minimal coverage of the length between the aligned portions of two proteins was applied to determine whether two CDS were members of the same gene family.

### Statistical and multivariate analyses

Quantitative data were presented as median and interquartile range with individual data points overlaid. Exploratory visualizations (barplots and boxplots) were produced using custom Python scripts (see Data availability, below).

To perform similarity groupings across the 40 isolates in each phenotypic parameter, Welch’s one-way analysis of variance (ANOVA) was used to account for unequal variances, followed by Games-Howell *post hoc* tests for pairwise comparisons (see Data availability, below). As the large number of isolates (*n* = 40) made traditional compact letter displays (CLD) difficult to interpret, pairwise Games-Howell *P*-values were used to construct greedy disjoint groups based on mutual non-significance. In the figures, letters a, b, c, etc. represent these similarity groups, and isolates were assigned the same letter only if they were not statistically significantly different (*P* > 0.05) from all other isolates in that letter group. While strains sharing a letter are statistically similar, strains with different letters are not necessarily significantly different from one another.

To facilitate standardized comparison and integrative visualization of phenotypic traits across diverse *Tenacibaculum* strains, averaged raw data from planktonic growth, macro-colony expansion, and submerged biofilm assays were independently rescaled to a range of 0–1 using min-max normalization (36):


(2) $$x_{rescaled}=\frac{x-x_{\min}}{x_{\max}-x_{\min}}$$

where *x* is the raw parameter value and *x*_rescaled_ is the normalized value. The resulting values were then inverted (1 - *x*_rescaled_) so that a score of 1 corresponded to the highest in that respective parameter and 0 to the lowest. By normalizing the data in this manner, the relative biological relevance was retained for each parameter while simplifying the heatmap interpretation. The heatmap was generated using a custom Python script (see Data availability, below).

Multivariate analyses were performed using a custom Python script (see Data availability, below). All phenotypic parameters were Z-score-normalized before principal component analysis (PCA). K-means clustering (with k = 4 based on the silhouette score) was applied to the first two principal components to partition strains into distinct phenotypic groups. Key visualizations generated from this analysis, including PCA scatter plots, correlation circles, and clustered heatmaps, are described in the figure legends.

CR/SYTO9 and TOTO-3/SYTO9 biovolume ratios were calculated using a custom Python script (see Data availability, below). The Python script performed statistical comparisons *via* an automated hypothesis-testing pipeline. Data normality was checked per group using the Shapiro-Wilk test, and homogeneity of variance was assessed *via* Levene’s test. For data sets satisfying normality assumptions but displaying unequal variances, Welch’s ANOVA was performed instead of one-way ANOVA, followed by the Games-Howell *post hoc* test for pairwise comparisons. Conversely, for data sets violating normality assumptions, group differences were evaluated using the nonparametric Kruskal-Wallis omnibus test followed by Dunn’s *post hoc* test with Holm’s adjustment.

Additional statistical comparisons were performed using independent *t*-tests with Welch’s correction (see Data availability, below). For variables deviating from normality, as determined by the Shapiro-Wilk test, the nonparametric Mann-Whitney U test was applied. Significance thresholds and notation followed the same criteria as described above. Statistical significance was determined at *P <* 0.05. The significance levels are denoted as follows: * for *P <* 0.05, ** for *P*  < 0.01, *** for *P* < 0.001, and **** for *P* < 0.0001.

## RESULTS

### Contrasting growth profiles emerge from planktonic growth kinetics in *Tenacibaculum*

The analysis revealed substantial phenotypic heterogeneity among the 40 strains, with growth kinetic parameters spanning a wide range. The planktonic growth rate (µ_max_) spanned an 8.2-fold range defined by the slowest-growing strain, *T. maritimum* CVI1001048 (TM14) with a rate of 0.095 h^−1^, and the fastest-growing strain, *T. maritimum* 190709D08d (TM9), which reached 0.778 h^−1^ (Fig. S2). Similarly, the planktonic N_max_ showed marked variability with a 3.5-fold difference between the lowest yield measured for TX (N_max_ of 0.302 OD_600_) and the highest yield in *T. litopenaei* LMG 23706T (TL; N_max_ of 1.043 OD_600_). For instance, TL exhibited a “high-yield, slow-growth” phenotype by pairing the highest recorded planktonic N_max_ with a low planktonic µmax of 0.098 h^−1^. Similarly, TM14 achieved a high final population density, reaching an N_max_ of 0.930 OD_600_, despite maintaining the minimum growth rate in the collection. In contrast, *T. agarivorans* HZ1T (TAG) showed an opposing “fast-growth, low-yield” phenotype, where a rapid growth rate of 0.709 h^−1^ was recorded, while reaching a significantly lower final biomass density with an N_max_ of 0.412 OD_600_. Furthermore, intraspecific heterogeneity was evident within *T. maritimum* as strains TM1 to TM19 spanned the full spectrum of observed growth rates in the data set.

### Broad spectrum of macro-colony phenotypes

**Fig 1:**
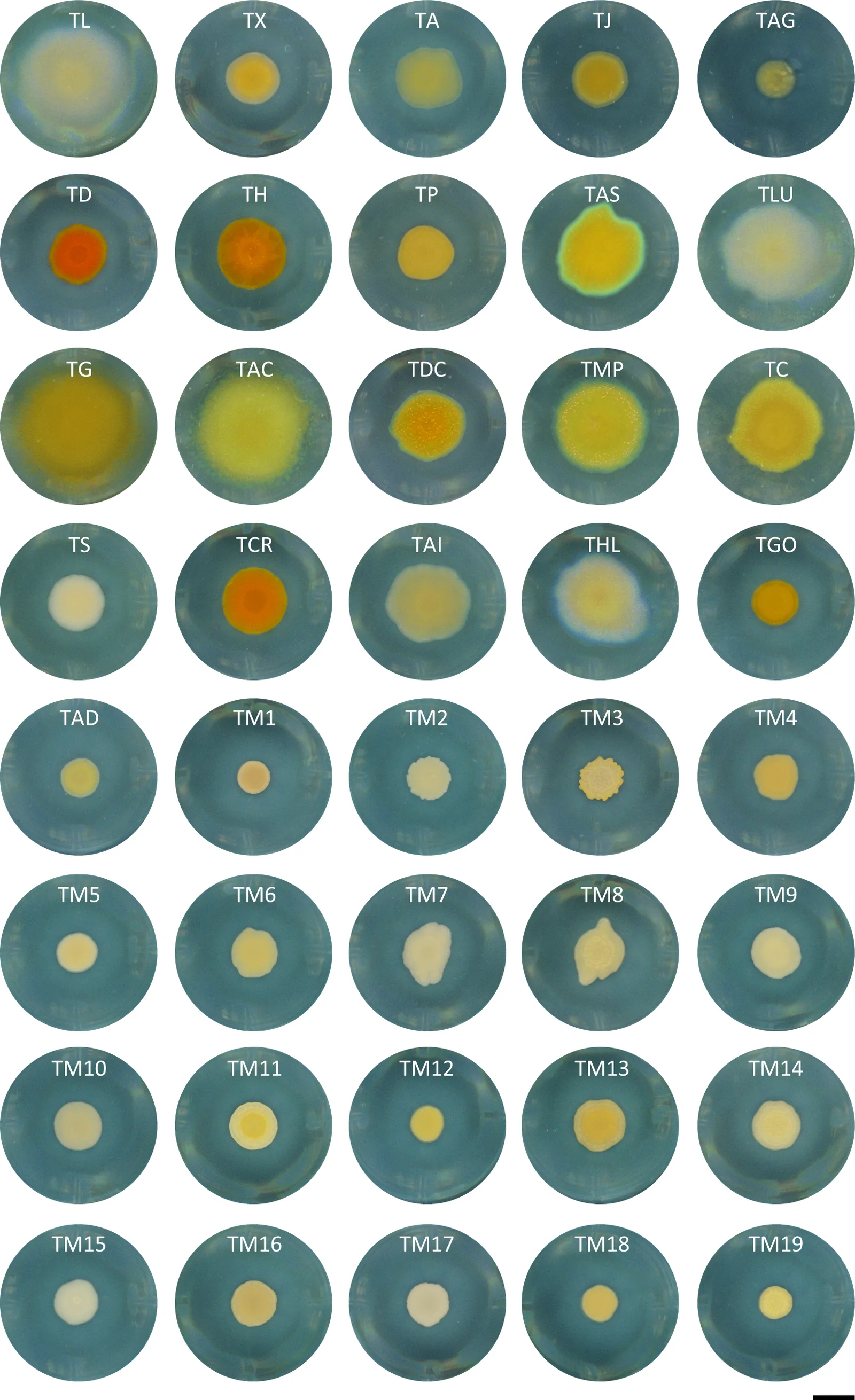
Phenotypic diversity of *Tenacibaculum* spp. macro-colony biofilms. Snapshot of 40 *Tenacibaculum* strains at 1 week of development in Marine Broth agar at 28°C. Scale bar = 5 mm.

Visual inspection of the macro-colony morphologies at 168 h highlighted a rich diversity, ranging from translucent, spreading layers to dense, highly pigmented, and circular shapes (Fig. 1). The visual color profiles of the macro-colonies are further represented by calculated mean HEX values from top- and bottom-illuminated images (Fig. S3). The apparent growth rate varied by more than 5-fold across the collection, ranging from 0.030 h^−1^ in TS, which corresponds to a doubling of colony surface area every 23.1 h, to 0.156 h^−1^ in TAG, which reached a doubling rate of every 4.4 h (Fig. S4). The apparent N_max_, representing the maximum final colony area, showed even greater heterogeneity, with a 15-fold difference between the smallest and the largest colonies. These boundaries were defined by *T. maritimum* FS081 (TM1), which produced a restricted colony area of 13.52 mm^2^, and *T. gallaicum* A37.1T (TG), which formed the largest macro-colony in the data set at 203.20 mm^2^. Significant intraspecific heterogeneity was maintained within *T. maritimum*, where the 19 strains spanned a 4.5-fold range in apparent µ_max_.

### Submerged biofilm maturation shows diverse architectural dynamics

**Fig 2:**
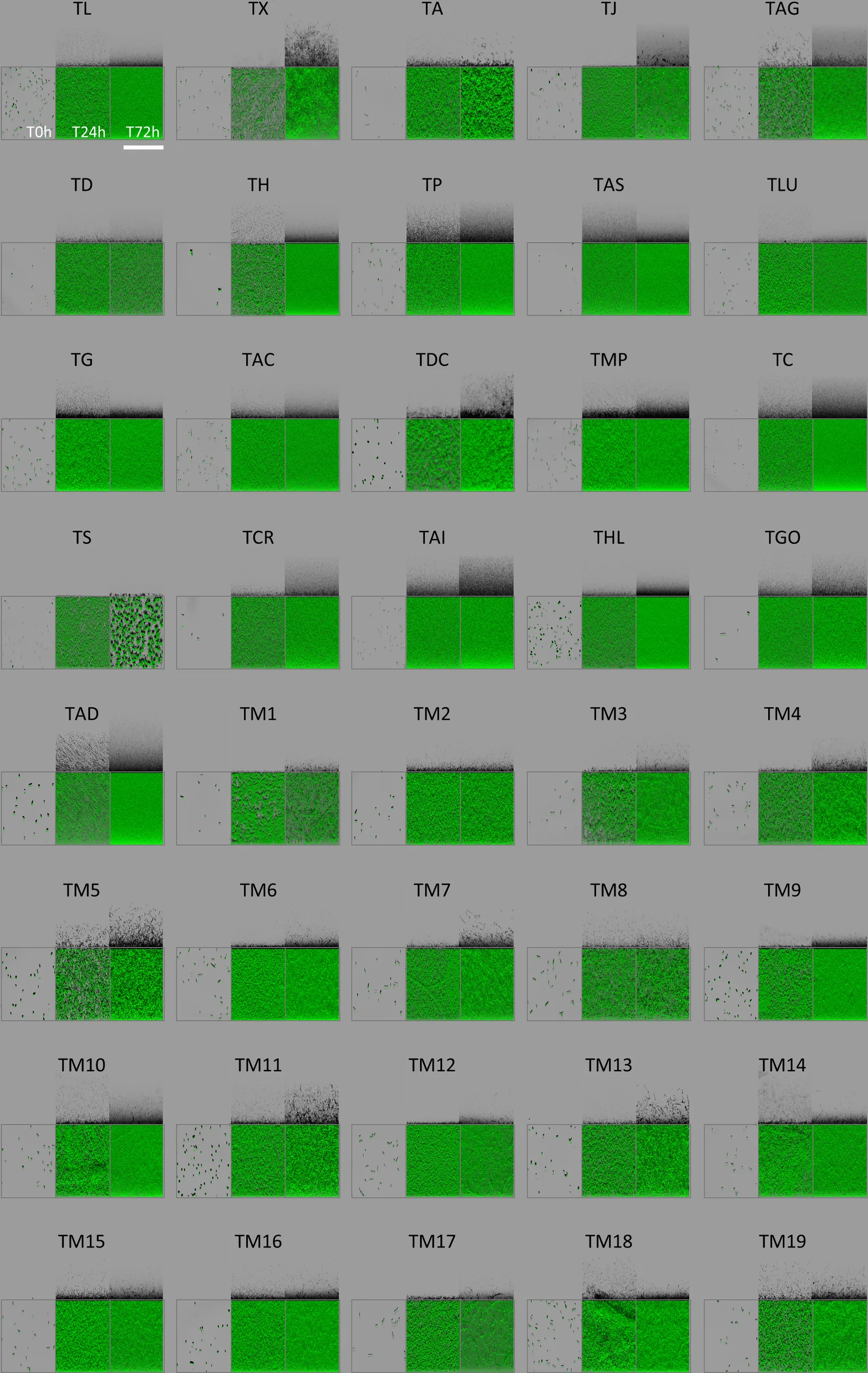
Diversity of submerged biofilm maturation dynamics in 40 *Tenacibaculum* strains. The biofilms were grown on 96-well plates in marine broth. For each of the 40 strains, observation snapshots were acquired at three specific developmental stages: T0h (initial cell adhesion), T24h (maturation phase), and T72h (late-stage development). Representative images from CLSM observations illustrate the structural evolution and architectural diversity of submerged biofilms across the collection. The 2D projections of the 3D biofilm architectures were generated from confocal z-stacks using IMARIS software in the blend mode. The artificial shadow is an xz section that provides a representation of the thickness and roughness of the biofilm. Scale bar = 40 µm.

Biofilm structural development of the strain collection at the surface-liquid interface was visualized using CLSM at T0h (i.e*.* after 1 h and 30 min of adhesion from planktonic culture), T24h, and T72h (Fig. 2). For each time point, total bacterial biovolumes were quantified (Fig. S5). T0h was performed to calibrate the initial adhesion immediately post-attachment. The recorded values were uniformly low, ranging from 0.0013 μm^3^/μm^2^ in *T. dicentrarchi* 3509T (TD) to 0.1166 μm^3^/μm^2^ in *T. maritimum* JIP 46/00 (TM19), confirming that biofilm formation was initiated from isolated cells rather than established clusters (Fig. S5). A substantial increase in biovolume was observed across the collection at T24h, indicating active maturation. At this stage, biovolume values spanned from 2.035 μm^3^/μm^2^ in TX to 40.646 μm^3^/μm^2^ in *T. aiptasiae* a4T (TAI). Further progression was noted for some strains at T72h, where the highest overall cellular biovolumes labeled with SYTO9 were recorded, extending from 2.030 μm^3^/μm^2^ in *T. sediminilitoris* KCTC 52210T (TS) to 57.098 μm^3^/μm^2^ in *T. caenipelagi* KCTC 32323T (TC). While many strains, such as TC, exhibited a 3.1-fold increase in biovolume between T24h and T72h, a reduction was observed in several others, including TAI and the clinical isolate *T. maritimum* TFA4 (TM8). Beyond cellular biovolume, other quantified spatial parameters provided insights into the morphological complexity of the submerged structures. Vertical development was characterized by maximum height and mean thickness (Fig. S6 and S7). Most strains continued to grow in these dimensions through T72h, with TC reaching a maximum mean thickness of 86.02 μm. Surface textural complexity, measured as roughness, showed intraspecific variability (Fig. S8). TAG displayed the highest surface roughness at 24 h, which was approximately 6.3-fold higher than that of TAI, the strain with the lowest roughness, whereas strains like TC showed a decrease in roughness at T72h by losing about 71.2% of its surface roughness compared to T24h, indicating a transition toward a more homogeneous, dense structure. Substratum coverage also varied, with many strains exceeding 0.9 a.u. by T72h, indicating coverage of almost all of the available surface area (Fig. S9).

Quantitative analysis of the extracellular matrix *via* CR-labeled matrix material biovolume further revealed the heterogeneous nature of matrix production within the collection (Fig. S10). At T24h, the volume of CR-labeled matrix material exhibited a wide range, from a minimum of 0.420 μm^3^/μm^2^ in TAG to a maximum of 8.643 μm^3^/μm^2^ in TMP. This diversity was intensified by T72h, where TAG shifted to the highest recorded matrix volume of 11.628 μm^3^/μm^2^, while *T. ascidiaceicola* KCTC 42702T (TAC) exhibited the lowest at 1.673 μm^3^/μm^2^.

To quantify matrix production compared to the abundance of bacterial cells, the matrix-to-biovolume ratio at 24 h was calculated (Fig. S11). The ratio remained low across the collection, with every isolate showing more cell volume compared to matrix volume with values below 0.5, except with TX, which exhibited more matrix material compared to the total cell volume with a ratio value of 2.65. Additionally, CR-labeled matrix material imaging (Fig. S12) revealed strain-specific differences in matrix organization, with notable variation in the degree of porosity, and spatial distribution within the biofilm.

### Multivariate integration of growth and structural traits identifies four phenotypic clusters

**Fig 3:**
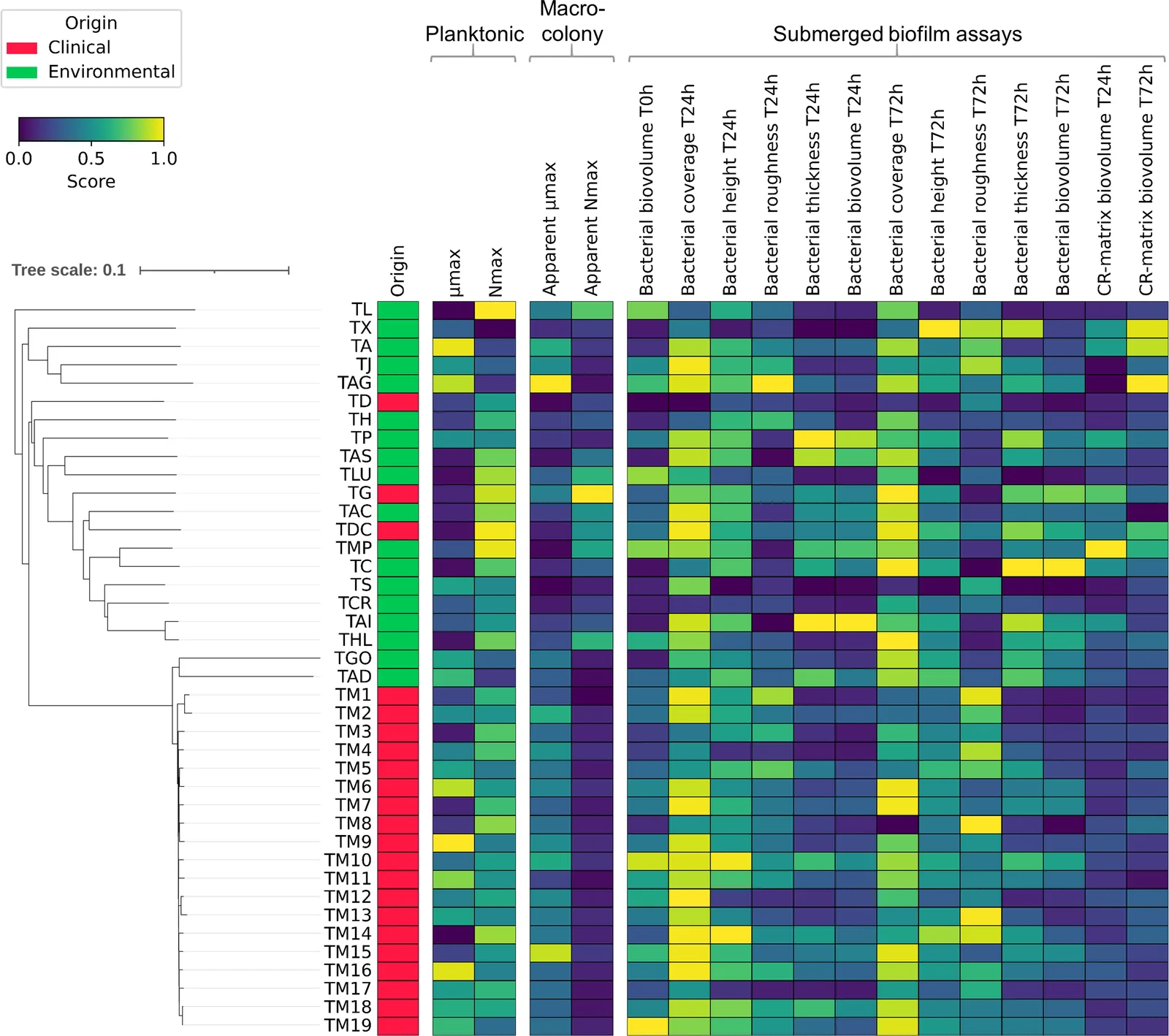
Heatmap of normalized phenotypic traits across *Tenacibaculum* strains. Phenotypic traits from planktonic growth (μ_max_ and N_max_), macro-colony expansion (apparent μ_max_ and apparent N_max_), and submerged biofilm assays (biovolume, mean thickness, height, roughness, substratum coverage, and Congo red-labeled matrix material) were independently min-max-normalized across strains and values inverted so that a score of 1 indicates the highest expression of a given trait and 0 the lowest. Strains are clustered based on the phylogenetic similarity (left dendrogram) and annotated with origin status: clinical (red) and environmental (green).

**Fig 4:**
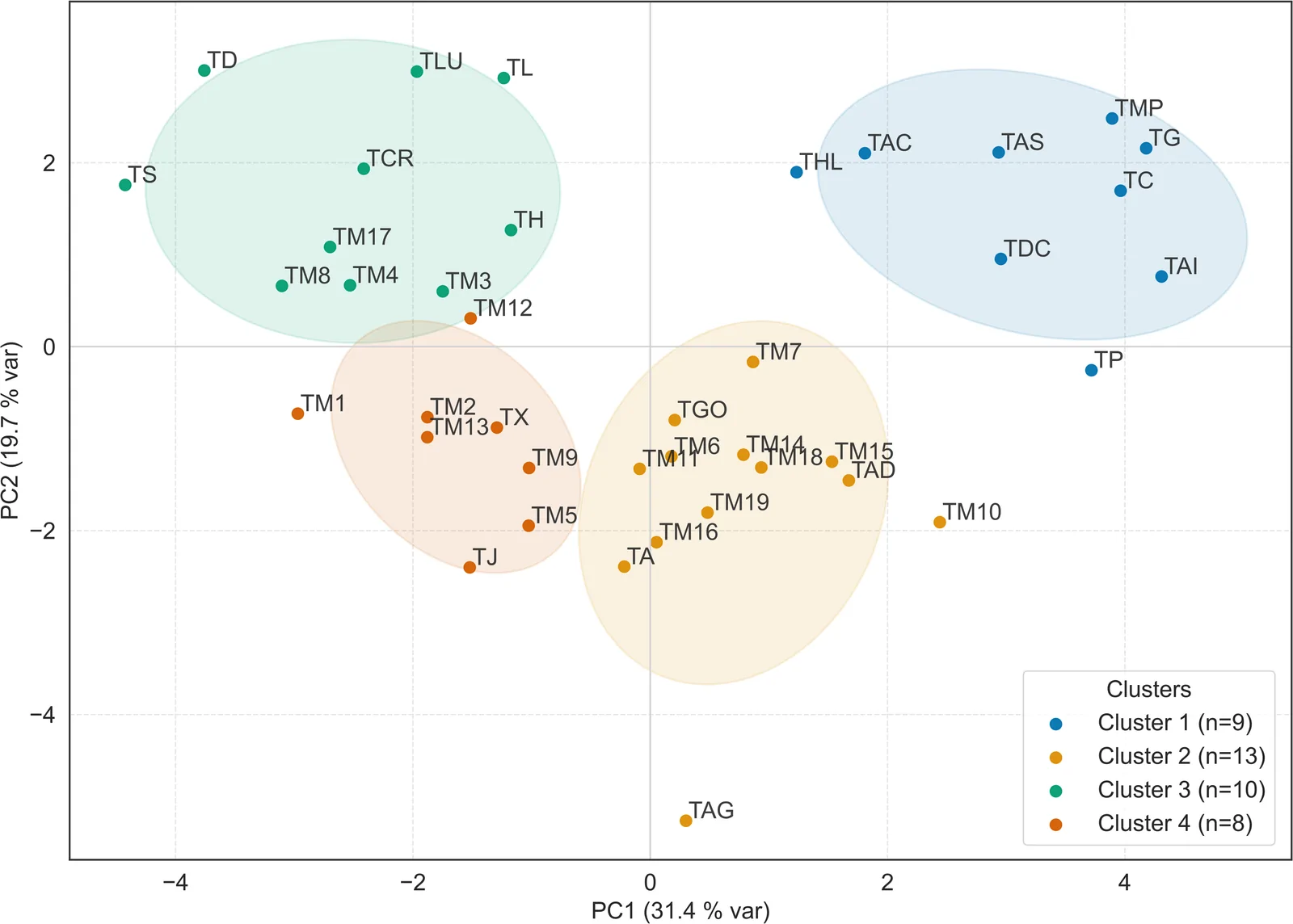
Principal component analysis (PCA) of averaged phenotypic traits across *Tenacibaculum* strains. Strains are projected onto the first two principal components derived from 17 phenotypic variables, accounting for 31.4% (PC1) and 19.7% (PC2) of the total variance. K-means clustering (k = 4 based on the silhouette score) was applied to the PCA scores, resulting in four distinct phenotypic clusters, each highlighted by color.

To provide an integrated overview of phenotypic diversity across the strain collection, the 17 phenotypic parameters were normalized and visualized as a heatmap ordered by phylogenetic similarity (Fig. 3). Based on the phylogenetic tree, all *T. martimum* strains (TM1–TM19) clustered at the basal portion of the tree. The heatmap provides visual cues of key phenotypic differences across both the planktonic and macro-colony models. *T. maritimum* strains trend toward higher growth rates with subsequent lower total biomass and colony area. Additionally, TM strains are distinguished by a lower abundance of CR-labeled matrix material. Further characterization of the phenotypic landscape of the *Tenacibaculum* collection was performed by integrating 17 phenotypic parameters into a multivariate analysis (Fig. 4). Following Z-score standardization of the data set to account for varying units and scales, principal component analysis (PCA) and K-means clustering were employed to identify functional groups based on the convergence of these growth and structural traits. The first two principal components, PC1 and PC2, accounted for 51.1% of the total variance of the data set, with PC1 contributing 31.4% and PC2 contributing 19.7%.

K-means clustering, performed on the first two principal components, partitioned the 40 strains into four distinct phenotypic groups (Fig. 4), each representing a unique phenotypic behavior. Cluster 1 was characterized by the most robust submerged biofilm structures, exhibiting the highest biovolumes and thickness values paired with a "high-yield, slow-growth" phenotype. In contrast, Cluster 2 contained the fastest-growing isolates in the collection and exhibited moderate architectural complexity. Cluster 3 was associated with the lowest submerged biofilm structural development while maintaining relatively substantial total biomass in liquid and high radial expansion in macro-colony models. Finally, Cluster 4 comprised strains with less-structured submerged biofilms and faster growth rates, but with a lower total population density.

Analysis of the PCA loading vectors (Fig. S13) further characterized the relationships between these phenotypic groups. A primary axis of variation was characterized by an anti-correlation between the growth rate and total biomass yield in planktonic culture; similarly in the macro-colony model, an anti-correlation was identified between the apparent growth rate and maximum colony size. Another anti-correlation was observed between CR-labeled matrix material at 24 h and biofilm roughness at 72 h. Conversely, biofilm roughness at 24 h was positively correlated with both planktonic and macro-colony growth rates. The majority of parameters associated with the submerged biofilm models displayed positive correlations. The distribution of strains across these clusters revealed significant intraspecific heterogeneity within *T. maritimum* as isolates were distributed across three of the four functional groups. A notable phylogenetic link was observed in Cluster 1 (Fig. S14), which consisted exclusively of non-*T. maritimum* species. In contrast, no distinct separation was observed between clinical and environmental isolates as both groups shared overlapping phenotypic profiles within the identified clusters.

### Clinical and environmental isolates show differences despite phenotypic overlap

To determine whether the origin of *Tenacibaculum* isolates is associated with differences in structural development, a comparative analysis was performed with the 17 phenotypic parameters between environmental and clinical isolates. While the broader architectural landscape showed substantial overlap, four specific parameters showed significant divergence by origin (Fig. S15). In submerged conditions, clinical isolates exhibited significantly higher biofilm roughness than environmental ones at T24h (*P* = 0.03). As the biofilms matured, this structural divergence became more pronounced at the T72h (*P* < 0.01). The macrocolony growth dynamics revealed significant differences in the primary kinetic parameters. Clinical isolates showed a significant increase in the apparent growth rate (*P* = 0.02). In contrast, environmental isolates reached a significantly higher apparent maximum growth area (*P* = 0.02).

### Divergent antibiotic tolerance across representative strains and their biofilms

**Fig 5:**
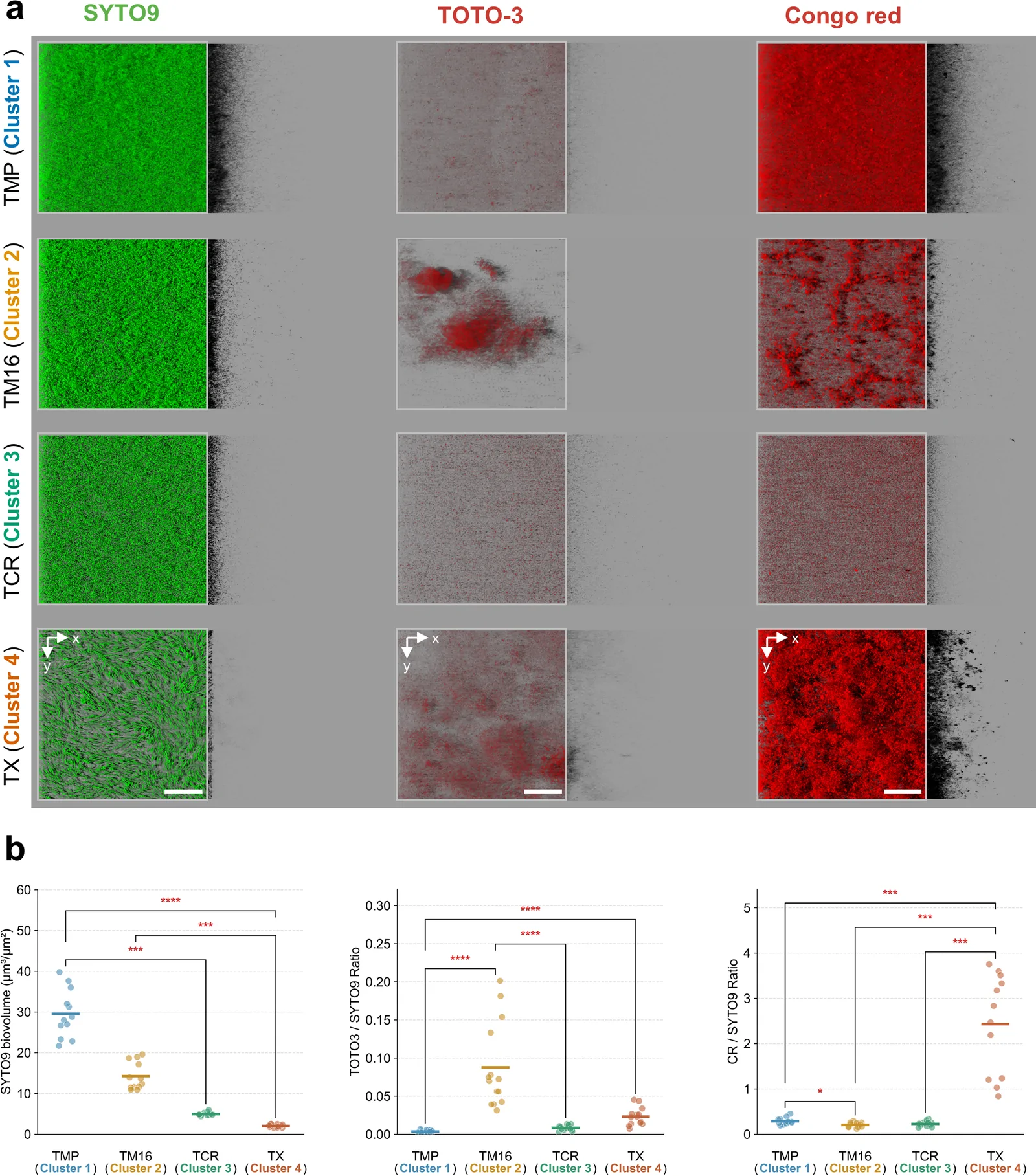
Biofilm structural characterization, eDNA, and matrix distribution of submerged biofilms at T24h formed by four representative *Tenacibaculum* strains belonging to four different clusters identified by multivariate analysis. (**a**) Strains TMP, TM16, TCR, and TX represent the structural variety and distinct phenotypic clusters identified across the collection. Biofilm structures are visualized using blend mode (2D projections) generated from confocal z-stacks *via* IMARIS software. Biofilms are stained to highlight bacterial biomass (green, SYTO9), eDNA (red, TOTO-3), and CR-labeled matrix material (red, CR). Scale bars = 40 µm. (**b**) Biofilm formation is quantified and represented as dot plots to display SYTO9 biovolume (µm³/µm²), ratio of eDNA, or CR-labeled matrix material compared to bacterial biomass. Statistical significance between strains was designated directly on the plots using bracket overlays with asterisks. The significance levels are denoted as follows: * for *P <* 0.05, ** for *P*  < 0.01, *** for *P* < 0.001, and **** for *P* < 0.0001.

To investigate the differences in antibiotic susceptibility between planktonic and biofilm lifestyles within the genus, four representative strains TMP, TM16, TCR, and TX (Fig. 5) were selected to study their capacity to tolerate antibiotics. The rationale for selecting these specific isolates was to capture a heterogeneous representation of the entire collection based on their bacterial biovolume (Fig. S5) and CR-labeled matrix biovolume at 24 h (Fig. S10). This selection was based on the wide range of matrix-to-biomass ratios (Fig. S11) and the four phenotypic clusters observed across the strains. Specifically, isolate TX was chosen for its low bacterial biovolume paired with high matrix production; others, such as TMP and TM16, were selected for their relatively high values for both parameters, and TCR was selected for its moderate biovolume with minimal matrix production.

Visual inspection of the 24-h biofilms in blend-mode projections revealed that while a common structural blueprint exists, the execution of biofilm structure varies significantly across the representative strains (Fig. 5a). In TMP, the bacterial biovolume was highest and more evenly distributed throughout the well than in other strains. The eDNA signal was notably scattered and appeared as isolated events with no palpable spatial organization. The isolate was embedded within a densely compact, non-porous CR-labeled matrix material. TCR had a moderate level of bacterial biomass and produced a minimal CR-labeled material that appeared only as a thin, discontinuous basal carpet with visible gaps. It displayed a scattered weak eDNA signal similar to TMP patterns. In contrast, TM16 harbored the most pronounced eDNA signal among the four strains. For the isolate, eDNA seemed concentrated in distinct patches that followed a spatially organized pattern. It was further accompanied by its visually evident vein-like matrix structures at the basal layer. TX displayed minimal bacterial biovolume with higher levels of eDNA and CR-labeled matrix material. The eDNA signal was sparser than the patterned aggregates of TM16. Interestingly, TX produced abundant CR-labeled matrix material that was well dispersed and highly porous.

Quantitative image analysis characterized bacterial biomass, eDNA, and CR-labeled materials across the four representative strains (Fig. 5b). Bacterial biovolume labeled by SYTO9 was compared among the strains at the 24-h time point, and TX displayed the lowest value, significantly lower than both TMP (*P* < 0.0001) and TM16 (*P* < 0.001). This aligns with its visually sparse cellular occupancy. TMP and TM16 were not significantly different from one another as both showed comparatively high bacterial biovolume. TCR showed an intermediate level that was significantly lower than that of TMP (*P* < 0.001) but not significantly different from that of TX (Fig. 5b). Next, quantification of the TOTO-3/SYTO9 biovolume ratio further revealed significant differences in the proportion of eDNA across the four representative strains. TM16 exhibited the highest ratio, and it significantly exceeded both TMP (*P* < 0.0001) and TCR (*P* < 0.0001). Similarly, TX harbored a high proportion of eDNA that was significantly higher than that of TMP (*P* < 0.0001). In essence, TCR occupied an intermediate position as it was not significantly different from either TMP or TX (Fig. 5b). In terms of the CR-labeled matrix material-to-biovolume ratio, TX exhibited an evidently escalated CR/SYTO9 ratio relative to all other strains (*P* < 0.001 vs TMP, TM16, and TCR). This was consistent with its visually apparent combination of minimal bacterial biomass and abundant matrix production (Fig. 5).

To assess the antimicrobial susceptibility of *Tenacibaculum* species, the MIC of OTC-HCl was determined for four representative strains (Fig. S16). The determined MIC values showed significant variation among the isolates, with TM16 demonstrating the highest sensitivity at 4 μg/mL, followed by TX at 32 μg/mL and TCR at 64 μg/mL, while TMP displayed a high baseline tolerance at 256 μg/mL.

Building upon these MIC values, a subsequent susceptibility experiment was conducted to determine whether the transition from a free-living state to a sessile lifestyle makes these bacteria significantly more resilient to treatment. The aim of this susceptibility assay was not to define clinical breakpoints but to compare planktonic and biofilm-associated survival under a standardized antimicrobial challenge with four representative strains. This comparison recapitulated the clinical challenge posed by established biofilms, which may contribute to the recurring failure of standard feed-based protocols despite the known planktonic sensitivity of the infecting strain.

**Fig 6:**
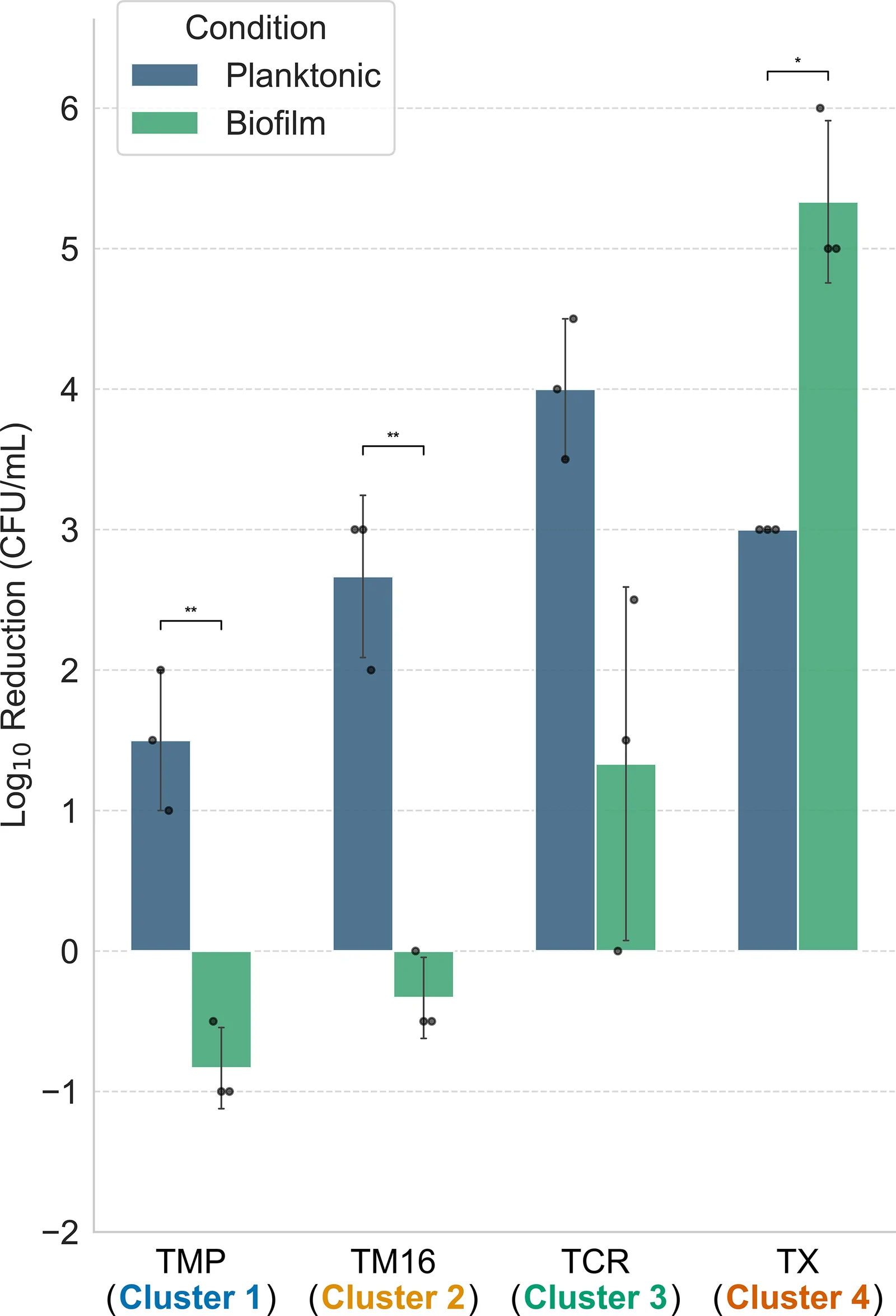
Comparative OTC-HCl susceptibility of representative *Tenacibaculum* strains in planktonic (blue) and biofilm (green) lifestyles. Logarithmic reduction in the viability of four representative *Tenacibaculum* strains (TM16, TX, TMP, and TCR) treated at respective 1× MIC. The chart compares susceptibility between planktonic and biofilm growth modes. Values represent the log10 reduction (CFU/mL) relative to untreated controls. The significance levels are denoted as follows: * for *P <* 0.05 and ** for *P*  < 0.01.

The results of this comparative assay demonstrated a profound shift in antibiotic susceptibility following the establishment of a 24-h biofilm. In the planktonic state, all isolates remained highly susceptible to the antibiotic at their respective MICs, with log reductions ranging from 1.50 to 4.00 CFU/mL (Fig. 6). However, the transition to a biofilm lifestyle conferred a substantial survival advantage for some strains. The most robust protection was observed in TM16 and TMP, which both demonstrated a statistically significant (*P* < 0.01) biofilm protection. The antibiotic effectively neutralized the planktonic form of TM16, resulting in a 2.67 log reduction. However, its transition to a biofilm state conferred complete protection, with the biofilm form exhibiting a reduction of −0.33 CFU/mL. Similarly, TMP, which had the highest recorded matrix biovolume, showed a total resistance to the antibiotic once it established a biofilm, recording a −0.83 log reduction. In contrast, TCR exhibited a notable numerical shift between growth states, moving from a 4.00 log reduction in its planktonic form to a 1.33 log reduction in the biofilm lifestyle. However, due to higher variability between biological replicates, this shift did not reach the threshold for statistical significance under Welch’s *t*-test. An opposite phenotype was exhibited by TX, although a significant difference was observed (*P* = 0.02), and its biofilm form was statistically more inhibited than its planktonic form.

## DISCUSSION

The characterization of 40 *Tenacibaculum* strains across planktonic, macrocolony, and submerged biofilm models reveals a sophisticated level of phenotypic diversity. Together, these findings indicate that biofilm structure in *Tenacibaculum* does not follow a single developmental trajectory but instead reflects strain-specific ecological profiles shaped by trade-offs between growth, biomass investment, and structural organization. Some traits were anticorrelated across models, whereas growth attributes, such as maximum growth rate and total biomass, showed consistent positive correlations between planktonic and macrocolony conditions. Strains such as TL follow a “high-yield, slow-growth” phenotype in both models, pairing low growth rates with high final biomass yields and large colony sizes. Conversely, clinical strains such as TM1–TM19 tend to show high growth rates but low biomass accumulation or final colony size. This acceleration may reflect rapid host exploitation, where the ability to quickly increase cell numbers during initial infection stages could be more advantageous than long-term density in a nutrient-limited environment (39). These results may suggest that both planktonic and macro-colony growth potential in *Tenacibaculum* reflect a trade-off between metabolic efficiency and proliferation rate, independent of the lifestyle or growing interface.

Macro-colony morphologies varied extensively, with pigmentation ranging from pale translucent yellow to vibrant orange-gold. This coloration is likely attributable to zeaxanthin, the primary carotenoid pigment in the genus, as *Tenacibaculum* species lack flexirubin-type pigments (5). This pigmentation may contribute, together with genomically encoded superoxide dismutases (14), to protect against reactive oxygen species in marine surface waters. Additionally, the heterogeneity in matrix production, quantified *via* CR-labeling, suggests that macro-colony morphology might be influenced by the quantity, biochemical composition, and spatial organization of the secreted matrix. Gliding motility is essential for the surface adaptation of this bacterium as *Tenacibaculum*, lacking flagella, actively colonizes the substratum by utilizing specialized machinery encoded by *gld* and *spr* genes that are associated with the T9SS (14, 40). The observed spectrum of dispersal phenotypes in macro-colonies may, therefore, be linked to differences in the production or activity of gliding-associated adhesins across strains. Furthermore, the diversity of colony morphologies observed across *Tenacibaculum* spp., from spreading translucent layers to dense circularized structures, may suggest the genus’s adaptation capacity to environmental variables, including different fish host species with varying mucous compositions, temperature fluctuations, and nutrient availability. Together, this phenotypic plasticity likely represents a critical evolutionary adaptation, allowing *Tenacibaculum* spp. to switch between rapid dispersal and sessile persistence depending on the environmental context, which perfectly embodies the genus’s etymology, *Tenacibaculum* (Latin *tenax*, “holding fast”), reflecting its tenacious nature (5).

In the submerged biofilm model, the transition from 24 to 72 h revealed a profound and heterogeneous remodeling of the community’s 3D spatial organization. While the biovolume generally increased, with TC reaching the highest value, several strains including the clinical isolate TM8 showed a notable reduction in biomass. This decrease in biomass at later time points may indicate active dispersal or structural remodeling, potentially to facilitate the colonization of new niches once local resources become limited (41). Alternative explanations such as cell death, detachment artifacts, or structural collapse cannot be excluded under the present experimental design (42). Another defining architectural feature identified across the *Tenacibaculum* collection is the highly variable spatial distribution of the extracellular matrix. Quantitative visualization of CR-labeled matrix material revealed that matrix components are not uniformly interspersed throughout the bacterial biomass, and for several isolates, they are densely sequestered at the biofilm-substratum interface. This basal enrichment of CR-labeled matrix material is consistent with the finding of a previous study showing that a robust adhesive base contributes to mechanical stability and supports subsequent vertical maturation (43). These CR-labeled materials are likely localized concentrations of specific functional polymers, such as amyloid-like proteins or β-linked polysaccharides (e.g., cellulose or chitin-like derivatives), which possess a high affinity for the diazo dye (32, 44). However, CR-positivity provides only a partial representation of the matrix as it specifically targets amyloid-like proteins or rigid β-structures (32). It is probable that a significant fraction of the matrix, including more hydrated, amorphous proteins, extracellular DNA (eDNA), and diverse heteropolysaccharides (45, 46), remains uncontrasted. A comprehensive characterization of the total matrix volume would necessitate the use of complementary labels such as fluorescently conjugated lectins and eDNA-intercalating stains (47, 48). Therefore, the observed spatial distribution should be interpreted as a proxy for specific matrix components rather than a comprehensive representation of total EPS.

Significant divergence in four specific parameters, including the apparent growth rate, apparent maximum growth area, and biofilm roughness at T24h and T72h, indicates functional distinctions between clinical and environmental *Tenacibaculum* strains. In submerged biofilm conditions, clinical isolates developed significantly higher surface roughness at 24 h. This phenotype was further accentuated at 72 h, when the difference in roughness between clinical and environmental isolates became even more pronounced. This reflects a transition toward more heterogeneous outer 3D spatial organization. Such elevated textural complexity mirrors phenotypes observed in pathogenic *Pseudomonas aeruginosa,* where increased roughness and structural peaks enhance nutrient access and antibiotic protection (49). From another perspective, higher roughness facilitates planktonic cell detachment from the biofilm (43). It has been described in S*almonella* that biofilm detachment releases planktonic cells that drive subsequent host pathogenesis (50). The significantly higher surface roughness observed in clinical *Tenacibaculum* isolates may similarly facilitate cell detachment, potentially contributing to host pathogenesis. Such notions suggest a putative link between *Tenacibaculum* biofilm morphology and pathogenic outcomes. However, definitive conclusions cannot be drawn with absolute confidence without further mechanistic studies. Besides, the clinical subset is dominated by *T. maritimum,* which may introduce taxonomic bias. These comparisons should, therefore, be interpreted as exploratory rather than definitive evidence of origin-specific adaptation.

The multivariate analysis of the 40 *Tenacibaculum* isolates revealed a phenotypic landscape defined by unique structural characteristics rather than taxonomic or origin-based divisions. The four clusters identified by PCA represent phenotypic groupings derived from multivariate structure rather than experimentally validated adaptive phenotypes. Delineated by unique biomarkers, Cluster 1 represents a “high-yield, slow-growth” phenotype that prioritizes the development of robust submerged biofilms characterized by maximum biovolume and thickness. In contrast, Cluster 2 comprises the fastest-growing isolates with moderate structural complexity. Cluster 3 is characterized by the lowest submerged biofilm structural development while maintaining high total biomass across both liquid and macro-colony models. Finally, Cluster 4 emphasizes kinetic speed over final density, yielding high growth rates but less complex 3D structures.

Four representative strains (TMP, TM16, TCR, and TX) spanning diverse biofilm morphologies were further studied to understand how structural divergences contribute to the observed failure of standard antimicrobial protocols. While representative strains showed broad planktonic sensitivity to OTC-HCl, establishment of biofilms eliminated such susceptibility in several strains. This phenotypic resilience is further validated by the findings of Tejero et al. (18), which reveal that while the skin mucus of gilthead seabream completely inhibited planktonic *T. maritimum* growth, it failed to disperse mature biofilms, highlighting the protective advantage of the sessile lifestyle against host antimicrobial defenses (18). In our study, TMP and TM16, with high bacterial and matrix biovolumes, manifested total recalcitrance. Both isolates shifted from multi-log reductions in liquid culture to effectively zero reduction once immobilized within the matrix. Though for TMP the eDNA-to-biomass ratio was lowest among the representative strains, the defensive barrier is likely driven by its highly saturated structural organization. This may limit antibiotic penetration or create local physicochemical gradients that reduce antimicrobial activity. While TM16 displayed a lower CR-labeled matrix material-to-biomass ratio, it harbored the highest eDNA proportions among other representative strains. These findings raise the possibility that, under conditions of high bacterial biomass, eDNA may compensate for a less dense matrix, thereby contributing to structural protection against OTC-HCl. Such notions are described in the literature as it is often shown that eDNA is a key structural scaffold in biofilm matrices that contribute to cohesion, surface adhesion, and tolerance to antimicrobial agents (51–53). The critical nature of this matrix-to-biomass and/or eDNA-to-biomass ratio is further highlighted by the divergence observed between strains TCR and TX. Most strikingly, the outlier phenotype of TX, where the biofilm was statistically more inhibited than its planktonic counterpart, despite having abundant CR-labeled matrix material, may reveal a structural vulnerability within the genus. Intensive investment in matrix material and the exceptionally low bacterial biovolume in TX appear to yield a porous, fragile biofilm that lacks the protective cellular density needed to impede antibiotic penetration. Together, the present data suggest an association between high bacterial biomass in combination with an elevated proportion of at least one of the matrix components, whereas eDNA or matrix material may confer greater protection against antibiotics. However, such findings do not directly resolve the underlying mechanisms. Future work combining spatial viability mapping, diffusion measurements, and further matrix compositional analysis will be required to disentangle the relative contributions of transport limitation, physiological heterogeneity, and structural shielding.

In addition, pan-genome analysis of *Tenacibaculum* strains (Table S2) highlights an exceptionally high level of genomic plasticity, with a reduced core genome (~610 CDS per strain) likely restricted to essential housekeeping functions such as DNA replication, transcription, and central metabolism (54). In contrast, the disproportionately large accessory genome, with up to 71.67% strain-specific CDS, underscores the evolutionary flexibility of this genus. This extensive genomic variability hints at the phenotypic diversity observed across the genus. Such an expanded accessory repertoire likely supports functional diversification and ecological adaptation, which may account for the broad phenotypic variability observed among *Tenacibaculum* strains. In this context, the relationship between the genotype and phenotype is unlikely to rely on single determinants but rather on multiple, potentially redundant or convergent genetic pathways. This genomic architecture may, therefore, enable distinct gene sets to drive similar phenotypic traits, particularly for complex functions such as virulence, as previously reported. Consequently, linking specific genetic markers to phenotypic outcomes in *Tenacibaculum* may require integrative approaches that go beyond gene presence/absence, incorporating gene regulation, community context, and environmental conditions. Ideally, experimental validation through targeted mutagenesis would be needed to functionally characterize molecular pathways, leading to biofilm formation. However, the lack of established genetic tools for *T. maritimum* and other species within the genus currently rules out such approaches (14).

### Conclusion

This study reveals extensive phenotypic diversity within the genus *Tenacibaculum* that transcends classical taxonomic boundaries, representing the first large-scale analysis combining such a number of strains and growth models. We demonstrate fundamental trade-offs between growth rate and biomass yield that are observed across planktonic and macro-colony models, which indicate intrinsic strain-specific growth phenotypes rather than model-dependent effects. In submerged biofilms, structural complexity and matrix material distribution vary, with strains exhibiting distinct temporal developmental dynamics. Furthermore, experiments on four representative strains indicate that the biofilm lifestyle can provide strain-dependent protection against oxytetracycline hydrochloride exposure. Together, by providing a tractable model system and a robust phenotypic data set, this work offers a foundation for future mechanistic studies of *Tenacibaculum* biofilm formation and for the development of biofilm-informed control strategies in aquaculture.

## Data Availability

Raw data sets, including image stacks from confocal microscopy, images and videos from macro-colony biofilms, and planktonic growth data for 40 *Tenacibaculum* spp. isolates, have been deposited in Data INRAE (accession number: https://doi.org/10.57745/GHNFYI). Further inquiries can be directed to the corresponding authors. Python codes for statistical analyses are accessible at https://forge.inrae.fr/sabit.ahmed/code-availability_tenaci/-/tree/1065e306a0c1bc0bb00e92eff3084f193fb0640f/.

## SUPPLEMENTAL MATERIAL

Supplemental material aem.01181-26-s0001.pdf Tables S1 and S2, Fig. S1 to S16, and legend for Video S1.

Video S1 aem.01181-26-s0002.mp4 Time-lapse monitoring of *Tenacibaculum* macro-colony development.

ASM does not own the copyrights to Supplemental Material that may be linked to, or accessed through, an article. The authors have granted ASM a non-exclusive, world-wide license to publish the Supplemental Material files. Please contact the corresponding author directly for reuse.

## References

[1] FAO. 2020. The state of world fisheries and aquaculture 2020. Sustainability in action. FAO, Rome, Italy.

[2] Escribano MP, Ramos-Pinto L, Fernández-Boo S, Afonso A, Costas B, Guardiola FA. 2020. Mucosal immune responses in senegalese sole (*Solea senegalensis*) juveniles after *Tenacibaculum maritimum* challenge: a comparative study between ocular and blind sides. Fish Shellfish Immunol 104:92–100. doi:10.1016/j.fsi.2020.05.080 32492465

[3] van Gelderen R, Carson J, Nowak B. 2011. Experimentally induced marine flexibacteriosis in Atlantic salmon smolts *Salmo salar*. II. pathology. Dis Aquat Organ 95:125–135. doi:10.3354/dao02329 21848120

[4] Mabrok M, Algammal AM, Sivaramasamy E, Hetta HF, Atwah B, Alghamdi S, Fawzy A, Avendaño-Herrera R, Rodkhum C. 2022. Tenacibaculosis caused by *Tenacibaculum maritimum*: updated knowledge of this marine bacterial fish pathogen. Front Cell Infect Microbiol 12:1068000. doi:10.3389/fcimb.2022.1068000 36683696 PMC9853564

[5] Duchaud E, Bernardet J-F. 2023. Tenacibaculum. In M.E. Trujillo, S. Dedysh, P. DeVos, B. Hedlund, P. Kämpfer, F.A. Rainey , W.B. Whitman (ed), Bergey’s Manual of Systematics of Archaea and Bacteria

[6] Avendaño-Herrera R, Irgang R, Sandoval C, Moreno-Lira P, Houel A, Duchaud E, Poblete-Morales M, Nicolas P, Ilardi P. 2016. Isolation, characterization and virulence potential of T*enacibaculum dicentrarchi* in salmonid cultures in chile. Transbound Emerg Dis 63:121–126. doi:10.1111/tbed.12464 26749435

[7] Tsertou MI, Triga A, Droubogiannis S, Kokkari C, Anasi G, Katharios P. 2023. Isolation and characterization of a novel *Tenacibaculum* species and a corresponding bacteriophage from a mediterranean fish hatchery: description of *Tenacibaculum larymnensis* sp. nov. and tenacibaculum phage larrie. Front Microbiol 14:1078669. doi:10.3389/fmicb.2023.1078669 36925475 PMC10013915

[8] Avendaño-Herrera R, Irgang R, Magariños B, Romalde JL, Toranzo AE. 2006. Use of microcosms to determine the survival of the fish pathogen T*enacibaculum maritimum* in seawater. Environ Microbiol 8:921–928. doi:10.1111/j.1462-2920.2005.00981.x 16623748

[9] Trivedi A, Miratsky JA, Henderson EC, Singharoy A, Shrivastava A. 2025. A membrane-associated conveyor belt controls the rotational direction of the bacterial type 9 secretion system. Available from: 10.1101/2024.09.23.614571 PMC12239597 40511966

[10] Bernardet J-F. 1998. *Cytophaga*, *Flavobacterium*, *Flexibacter* and *Chryseobacterium* infections in cultured marine fish. Fish Pathol 33:229–238. doi:10.3147/jsfp.33.229

[11] Ferguson HW, Delannoy CMJ, Hay S, Nicolson J, Sutherland D, Crumlish M. 2010. Jellyfish as vectors of bacterial disease for farmed salmon (*Salmo salar*). J Vet Diagn Invest 22:376–382. doi:10.1177/104063871002200305 20453210

[12] Wakabayashi H, Hikida M, Masumura K, Wakabayashi H, Hikida M, Masumura K, Wakabayashi H, Hikida M, Masumura K. 1986. *Flexibacter maritimus* sp. nov., a pathogen of marine fishes. Int J Syst Bacteriol 36:396–398. doi:10.1099/00207713-36-3-396

[13] Suzuki M, Nakagawa Y, Harayama S, Yamamoto S. 2001. Phylogenetic analysis and taxonomic study of marine *Cytophaga*-like bacteria: proposal for *Tenacibaculum* gen. nov. with *Tenacibaculum maritimum* comb. nov. and *Tenacibaculum ovolyticum* comb. nov., and description of *Tenacibaculum mesophilum* sp. nov. and *Tenacibaculum amylolyticum* sp. nov. Int J Syst Evol Microbiol 51:1639–1652. doi:10.1099/00207713-51-5-1639 11594591

[14] Pérez-Pascual D, Lunazzi A, Magdelenat G, Rouy Z, Roulet A, Lopez-Roques C, Larocque R, Barbeyron T, Gobet A, Michel G, Bernardet J-F, Duchaud E. 2017. The complete genome sequence of the fish pathogen *Tenacibaculum maritimum* provides insights into virulence mechanisms. Front Microbiol 8:1542. doi:10.3389/fmicb.2017.01542 28861057 PMC5561996

[15] Nadell CD, Drescher K, Foster KR. 2016. Spatial structure, cooperation and competition in biofilms. Nat Rev Microbiol 14:589–600. doi:10.1038/nrmicro.2016.84 27452230

[16] Sauer K, Stoodley P, Goeres DM, Hall-Stoodley L, Burmølle M, Stewart PS, Bjarnsholt T. 2022. The biofilm life cycle: expanding the conceptual model of biofilm formation. Nat Rev Microbiol 20:608–620. doi:10.1038/s41579-022-00767-0 35922483 PMC9841534

[17] Flemming H-C, Wuertz S. 2019. Bacteria and archaea on earth and their abundance in biofilms. Nat Rev Microbiol 17:247–260. doi:10.1038/s41579-019-0158-9 30760902

[18] Tejero M, Sanahuja I, Balsalobre C, Ibarz A, Madrid C, Fernandez-Alacid L. 2025. Biofilm formation of *Tenacibaculum maritimum*, a fish pathogenic bacteria, to evaluate the antimicrobial activity of fish skin mucus. Front Mar Sci 12:1631980. doi:10.3389/fmars.2025.1631980

[19] Baxa DV, Kawai K, Kusuda R. 1988. *In vitro* and *in vivo* activities of *Flexibacter maritimus* toxins. Rep Usa Mar Biol Inst Kochi Univ 10:1–8.

[20] Van Gelderen R, Carson J, Gudkovs N, Nowak B. 2010. Physical characterisation of *Tenacibaculum maritimum* for vaccine development. J Appl Microbiol 109:1668–1676. doi:10.1111/j.1365-2672.2010.04795.x 20636343

[21] Levipan HA, Irgang R, Tapia-Cammas D, Avendaño-Herrera R. 2019. A high-throughput analysis of biofilm formation by the fish pathogen *Tenacibaculum dicentrarchi*. J Fish Dis 42:617–621. doi:10.1111/jfd.12949 30664803

[22] Levipan HA, Tapia-Cammas D, Molina V, Irgang R, Toranzo AE, Magariños B, Avendaño-Herrera R. 2019. Biofilm development and cell viability: an undervalued mechanism in the persistence of the fish pathogen *Tenacibaculum maritimum*. Aquaculture 511:734267. doi:10.1016/j.aquaculture.2019.734267

[23] Bondad‐Reantaso MG, MacKinnon B, Karunasagar I, Fridman S, Alday‐Sanz V, Brun E, Le Groumellec M, Li A, Surachetpong W, Karunasagar I, Hao B, Dall’Occo A, Urbani R, Caputo A. 2023. Review of alternatives to antibiotic use in aquaculture. Rev Aquac 15:1421–1451. doi:10.1111/raq.12786

[24] Bridel S, Bourgeon F, Marie A, Saulnier D, Pasek S, Nicolas P, Bernardet J-F, Duchaud E. 2020. Genetic diversity and population structure of *Tenacibaculum maritimum*, a serious bacterial pathogen of marine fish: from genome comparisons to high throughput MALDI-TOF typing. Vet Res 51:60. doi:10.1186/s13567-020-00782-0 32381115 PMC7204230

[25] Lopez P, Bridel S, Saulnier D, David R, Magariños B, Torres BS, Bernardet JF, Duchaud E. 2022. Genomic characterization of *Tenacibaculum maritimum* O‐antigen gene cluster and development of a multiplex PCR‐based serotyping scheme. Transbounding Emerging Dis 69:e2876–e2888. doi:10.1111/tbed.14637 PMC9796276 35731505

[26] Ondov BD, Treangen TJ, Melsted P, Mallonee AB, Bergman NH, Koren S, Phillippy AM. 2016. Mash: fast genome and metagenome distance estimation using MinHash. Genome Biol 17:132. doi:10.1186/s13059-016-0997-x 27323842 PMC4915045

[27] Serra DO, Klauck G, Hengge R. 2015. Vertical stratification of matrix production is essential for physical integrity and architecture of macrocolony biofilms of *Escherichia coli*. Environ Microbiol 17:5073–5088. doi:10.1111/1462-2920.12991 26234179 PMC5014196

[28] Arnaouteli S, Bamford NC, Stanley-Wall NR, Kovács ÁT. 2021. *Bacillus subtilis* biofilm formation and social interactions. Nat Rev Microbiol 19:600–614. doi:10.1038/s41579-021-00540-9 33824496

[29] Gingichashvili S, Steinberg D, Sionov RV, Feuerstein O, Cohen NE. 2022. An open-source computational tool for measuring bacterial biofilm morphology and growth kinetics upon one-sided exposure to an antimicrobial source. Sci Rep 12:16125. doi:10.1038/s41598-022-20275-8 36167741 PMC9515175

[30] Serra DO, Richter AM, Klauck G, Mika F, Hengge R. 2013. Microanatomy at cellular resolution and spatial order of physiological differentiation in a bacterial biofilm. mBio 4:e00103-13. doi:10.1128/mBio.00103-13 23512962 PMC3604763

[31] Baranyi J, Roberts TA. 1994. A dynamic approach to predicting bacterial growth in food. Int J Food Microbiol 23:277–294. doi:10.1016/0168-1605(94)90157-0 7873331

[32] Jones CJ, Wozniak DJ. 2017. Congo red stain identifies matrix overproduction and is an indirect measurement for c-di-GMP in many species of bacteria. Methods Mol Biol 1657:147–156. doi:10.1007/978-1-4939-7240-1_12 28889292

[33] Sillesen FW, Dicke F, Kath-Schorr S, Weissinger H, Kjems J, Minero GAS, Meyer R. 2026. Unmasking the diversity of extracellular nucleic acids in the biofilm matrix using nucleic acid-binding dyes. bioRxiv. doi:10.64898/2026.05.08.723897

[34] Hartmann R, Jeckel H, Jelli E, Singh PK, Vaidya S, Bayer M, Rode DKH, Vidakovic L, Díaz-Pascual F, Fong JCN, Dragoš A, Lamprecht O, Thöming JG, Netter N, Häussler S, Nadell CD, Sourjik V, Kovács ÁT, Yildiz FH, Drescher K. 2021. Quantitative image analysis of microbial communities with BiofilmQ. Nat Microbiol 6:151–156. doi:10.1038/s41564-020-00817-4 33398098 PMC7840502

[9] Guéneau V, Charron R, Costache V, Bridier A, Briandet R. 2023. Spatial analysis of multispecies bacterial biofilms, p 275–307. In Methods in Microbiology. Vol. 53. Academic Press.

[36] Guéneau V, Guillier L, Berdous C, Noirot-Gros M-F, Jiménez G, Plateau-Gonthier J, Serror P, Castex M, Briandet R. 2025. 3D imaging-driven assembly of multispecies biofilms with antagonistic activity against undesirable bacteria. ISME Commun 5:ycaf156. doi:10.1093/ismeco/ycaf156 41127251 PMC12539569

[37] Vallenet D, Engelen S, Mornico D, Cruveiller S, Fleury L, Lajus A, Rouy Z, Roche D, Salvignol G, Scarpelli C, Médigue C. 2009. MicroScope: a platform for microbial genome annotation and comparative genomics. Database (Oxford) 2009:bap021. doi:10.1093/database/bap021 20157493 PMC2790312

[38] Lopez P, Fradet B, Coffion L, Bernardet J-F, Saulnier D, Duchaud E. 2025. *Tenacibaculum platacis* sp. nov., *Tenacibaculum vairaonense* sp. nov. and *Tenacibaculum polynesiense* sp. nov. isolated from batfish (*Platax orbicularis*) in Tahiti Island, French Polynesia. Int J Syst Evol Microbiol 75. doi:10.1099/ijsem.0.006605 PMC12281758 39757983

[39] Didelot X, Walker AS, Peto TE, Crook DW, Wilson DJ. 2016. Within-host evolution of bacterial pathogens. Nat Rev Microbiol 14:150–162. doi:10.1038/nrmicro.2015.13 26806595 PMC5053366

[40] Avendaño-Herrera R, Echeverría-Bugueño M, Hernández M, Saldivia P, Irgang R. 2024. Proteomic characterization of *Tenacibaculum dicentrarchi* under iron limitation reveals an upregulation of proteins related to iron oxidation and reduction metabolism, iron uptake systems and gliding motility. J Fish Dis 47:e13984. doi:10.1111/jfd.13984 38943549

[41] McDougald D, Rice SA, Barraud N, Steinberg PD, Kjelleberg S. 2012. Should we stay or should we go: mechanisms and ecological consequences for biofilm dispersal. Nat Rev Microbiol 10:39–50. doi:10.1038/nrmicro2695 22120588

[42] Dergham Y, Le Coq D, Nicolas P, Bidnenko E, Dérozier S, Deforet M, Huillet E, Sanchez-Vizuete P, Deschamps J, Hamze K, Briandet R. 2023. Direct comparison of spatial transcriptional heterogeneity across diverse *Bacillus subtilis* biofilm communities. Nat Commun 14:7546. doi:10.1038/s41467-023-43386-w 37985771 PMC10661151

[43] Wang S, Zhu H, Zheng G, Dong F, Liu C. 2022. Dynamic changes in biofilm structures under dynamic flow conditions. Appl Environ Microbiol 88:e0107222. doi:10.1128/aem.01072-22 36300948 PMC9680615

[44] Thongsomboon W, Werby SH, Cegelski L. 2020. Evaluation of phosphoethanolamine cellulose production among bacterial communities using congo red fluorescence. J Bacteriol 202:e00030-20. doi:10.1128/JB.00030-20 32312746 PMC7283597

[45] Costerton JW, Stewart PS, Greenberg EP. 1999. Bacterial biofilms: a common cause of persistent infections. Science 284:1318–1322. doi:10.1126/science.284.5418.1318 10334980

[46] Flemming H-C, Wingender J. 2010. The biofilm matrix. Nat Rev Microbiol 8:623–633. doi:10.1038/nrmicro2415 20676145

[47] Okshevsky M, Meyer RL. 2014. Evaluation of fluorescent stains for visualizing extracellular DNA in biofilms. J Microbiol Methods 105:102–104. doi:10.1016/j.mimet.2014.07.010 25017901

[48] Neu TR, Kuhlicke U. 2022. Matrix glycoconjugate characterization in multispecies biofilms and bioaggregates from the environment by means of fluorescently-labeled lectins. Front Microbiol 13:940280. doi:10.3389/fmicb.2022.940280 36003926 PMC9395170

[49] Thi MTT, Wibowo D, Rehm BHA. 2020. *Pseudomonas aeruginosa* biofilms. IJMS 21:8671. doi:10.3390/ijms21228671 33212950 PMC7698413

[50] Hamilton S, Bongaerts RJM, Mulholland F, Cochrane B, Porter J, Lucchini S, Lappin-Scott HM, Hinton JCD. 2009. The transcriptional programme of *Salmonella enterica* serovar typhimurium reveals a key role for tryptophan metabolism in biofilms. BMC Genomics 10:599. doi:10.1186/1471-2164-10-599 20003355 PMC2805695

[51] Okshevsky M, Regina VR, Meyer RL. 2015. Extracellular DNA as a target for biofilm control. Curr Opin Biotechnol 33:73–80. doi:10.1016/j.copbio.2014.12.002 25528382

[52] Okshevsky M, Meyer RL. 2015. The role of extracellular DNA in the establishment, maintenance and perpetuation of bacterial biofilms. Crit Rev Microbiol 41:341–352. doi:10.3109/1040841X.2013.841639 24303798

[53] Panlilio H, Rice CV. 2021. The role of extracellular DNA in the formation, architecture, stability, and treatment of bacterial biofilms. Biotechnol Bioeng 118:2129–2141. doi:10.1002/bit.27760 33748946 PMC8667714

[54] Segata N, Huttenhower C. 2011. Toward an efficient method of identifying core genes for evolutionary and functional microbial phylogenies. PLoS One 6:e24704. doi:10.1371/journal.pone.0024704 21931822 PMC3171473

