# Supplementary material for: Diversity of biofilm architectures within the genus Tenacibaculum
Source: Appl Environ Microbiol. 2026 Jul 31;92(8):e01181-26. doi: 10.1128/aem.01181-26 (PMC13488331; doi:10.1128/aem.01181-26)
Supplement: Supplementary file 1 [file aem.01181-26-s0001.pdf]

Supplementary Tables

Sup. table 1: Characteristics of bacterial strains used in this study.

Primary identifier	Strain code	Name	Origin	RefSeq accession No.
TL	27	*T. litopenaei* LMG 23706T	Environmental	GCF_964514025.1
TX	3	*T. xiamenense* LMG 27422T	Environmental	GCF_964513065.1
TA	19	*T. amylolyticum* CIP 107214T	Environmental	GCF_964511145.1
TJ	4	*T. jejuense* KCTC 22618T	Environmental	GCF_900198195.1
TAG	5	*T. agarivorans* HZ1T	Environmental	GCF_001936575.1
TD	7	*T. dicentrarchi* 3509T	Clinical	GCF_964036635.1
TH	26	*T. holothuriorum* LMG27758T	Environmental	GCF_002120225.1
TP	2	*T. polynesiense* 190130A14aT	Environmental	GCF_964048965.1
TAS	17	*T. aestuarii* SMK4T	Environmental	GCF_964510395.1
TLU	28	*T. lutimaris* DSM 16505T	Environmental	GCF_003610735.1
TG	23	*T. gallaicum* A37.1T	Clinical	GCF_003387615.1
TAC	20	*T. ascidiaceicola* KCTC 42702T	Environmental	GCF_964512845.1
TDC	1	*T. discolor* LL04 11.1.1T	Clinical	GCF_003664185.1
TMP	29	*T. mesophilum* CIP 107215T	Environmental	GCF_009362255.1
TC	21	*T. caenipelagi* KCTC 32323T	Environmental	GCF_004363005.1
TS	30	*T. sediminilitoris* KCTC 52210T	Environmental	GCF_964513795.1
TCR	22	*T. crassostreae* JO1T	Environmental	GCF_964512405.1
TAI	18	*T. aiptasiae* a4T	Environmental	GCF_008806755.1
THL	25	*T. halocynthiae* KCTC 32262T	Environmental	GCF_964512825.1
TGO	24	*T. geojense* YCS6T	Environmental	GCF_042676005.1
TAD	16	*T. adriaticum* DSM 18961T	Environmental	GCF_008124875.1
TM1	13	*T. maritimum* FS081	Clinical	GCF_902705395.1
TM2	42	*T. maritimum* NAC SLCC MFF	Clinical	GCF_902705345.1
TM3	6	*T. maritimum* NCIMB 2154T	Clinical	GCF_900119795.1
TM4	8	*T. maritimum* NCIMB 2158	Clinical	GCF_902705425.1
TM5	37	*T. maritimum* JIP 32/91-4	Clinical	GCF_902705385.1
TM6	34	*T. maritimum* P4-45	Clinical	GCF_902705495.1
TM7	14	*T. maritimum* DPIF 89/0239-1	Clinical	GCF_902705355.1
TM8	9	*T. maritimum* TFA4	Clinical	GCF_902705565.1
TM9	40	*T. maritimum* 190709D08d	Clinical	pubmlst.org (id=152)*
TM10	31	*T. maritimum* FC	Clinical	GCA_902705415.1
TM11	38	*T. maritimum* UCD SB2	Clinical	GCF_902705445.1
TM12	36	*T. maritimum* JIP 10/97	Clinical	GCA_902705285.1
TM13	41	*T. maritimum* 190115A05h	Clinical	pubmlst.org (id=150)*
TM14	12	*T. maritimum* CVI1001048	Clinical	GCF_902705265.1
TM15	32	*T. maritimum* Aq16-89	Clinical	GCA_902705375.1
TM16	39	*T. maritimum* 190605A03b	Clinical	pubmlst.org (id=151)*
TM17	10	*T. maritimum* 190628A06a	Clinical	pubmlst.org (id=153)*
TM18	43	*T. maritimum* P1-39	Clinical	GCA_902705535.1
TM19	35	*T. maritimum* JIP 46/00	Clinical	GCA_902705435.1

* genome available in PubMLST (https://pubmlst.org/bigsdb?db=pubmlst_tenacibaculum_isolates&page=query).
Isolate id is given in parentheses.

Sup. table 2: Pan-genome analysis of 40 *Tenacibaculum* strains. The pan-genome is made up of 38,138 gene families totaling 122,377 genes. Here, 606 families (24,417 genes) were shared by all strains and constitute the core-genome. The variable genome had 37,532 families (97,960 genes). For each strain, the total number of CDS, their distribution across core, variable, and strain-specific compartments, and the corresponding percentages are detailed below. A cutoff of 80% identity and 80% on the minimal coverage of the length between the aligned portions of two proteins was applied to determine whether two CDS were members of the same gene family.

Primary identifier	CDS	CDS (without artefact fam.)	Pan CDS	Core CDS	Var CDS	Strain specific CDS	Core CDS (%)	Var CDS (%)	Strain spe. CDS (%)	Excluded CDS (%)
TL	4044	4031	4031	610	3421	2889	15.133	84.867	71.67	0
TX	4456	4452	4452	612	3840	2970	13.747	86.253	66.712	0
TA	4053	4042	4042	609	3433	2515	15.067	84.933	62.222	0
TJ	3969	3955	3955	610	3345	2245	15.424	84.576	56.764	0
TAG	4731	4705	4705	609	4096	2966	12.944	87.056	63.039	0
TD	2354	2347	2347	612	1735	957	26.076	73.924	40.775	0
TH	2949	2935	2935	611	2324	1365	20.818	79.182	46.508	0
TP	3579	3570	3570	611	2959	1701	17.115	82.885	47.647	0
TAS	2747	2735	2735	610	2125	374	22.303	77.697	13.675	0
TLU	2689	2653	2653	610	2043	294	22.993	77.007	11.082	0
TG	3137	3098	3098	611	2487	391	19.722	80.278	12.621	0
TAC	3226	3164	3164	612	2552	311	19.343	80.657	9.829	0
TDC	3160	3127	3127	610	2517	272	19.508	80.492	8.698	0
TMP	2992	2965	2965	610	2355	417	20.573	79.427	14.064	0
TC	3124	3099	3099	610	2489	830	19.684	80.316	26.783	0
TS	2770	2759	2759	611	2148	569	22.146	77.854	20.623	0
TCR	2457	2444	2444	611	1833	561	25	75	22.954	0
TAI	3599	3579	3579	611	2968	1709	17.072	82.928	47.751	0
THL	3851	3836	3836	611	3225	2235	15.928	84.072	58.264	0
TGO	2655	2645	2645	611	2034	1524	23.1	76.9	57.618	0
TAD	2998	2981	2981	610	2371	1731	20.463	79.537	58.068	0
TM1	3010	2886	2886	611	2275	168	21.171	78.829	5.821	0
TM2	2973	2822	2822	610	2212	130	21.616	78.384	4.607	0
TM3	2978	2791	2791	610	2181	88	21.856	78.144	3.153	0
TM4	2963	2822	2822	612	2210	88	21.687	78.313	3.118	0
TM5	3028	2893	2893	610	2283	69	21.085	78.915	2.385	0
TM6	2968	2867	2867	610	2257	113	21.277	78.723	3.941	0
TM7	3157	2836	2836	610	2226	85	21.509	78.491	2.997	0
TM8	2990	2880	2880	610	2270	70	21.181	78.819	2.431	0
TM9	2865	2749	2749	611	2138	25	22.226	77.774	0.909	0
TM10	3092	2955	2955	610	2345	171	20.643	79.357	5.787	0

TM11	2840	2735	2735	610	2125	54	22.303	77.697	1.974	0
TM12	2898	2765	2765	610	2155	65	22.061	77.939	2.351	0
TM13	2977	2719	2719	610	2109	54	22.435	77.565	1.986	0
TM14	2784	2684	2684	610	2074	29	22.727	77.273	1.08	0
TM15	2761	2669	2669	610	2059	10	22.855	77.145	0.375	0
TM16	2835	2731	2731	610	2121	4	22.336	77.664	0.146	0
TM17	3065	2859	2859	610	2249	151	21.336	78.664	5.282	0
TM18	2834	2796	2796	611	2185	56	21.853	78.147	2.003	0
TM19	2902	2796	2796	610	2186	64	21.817	78.183	2.289	0

Supplementary Figures

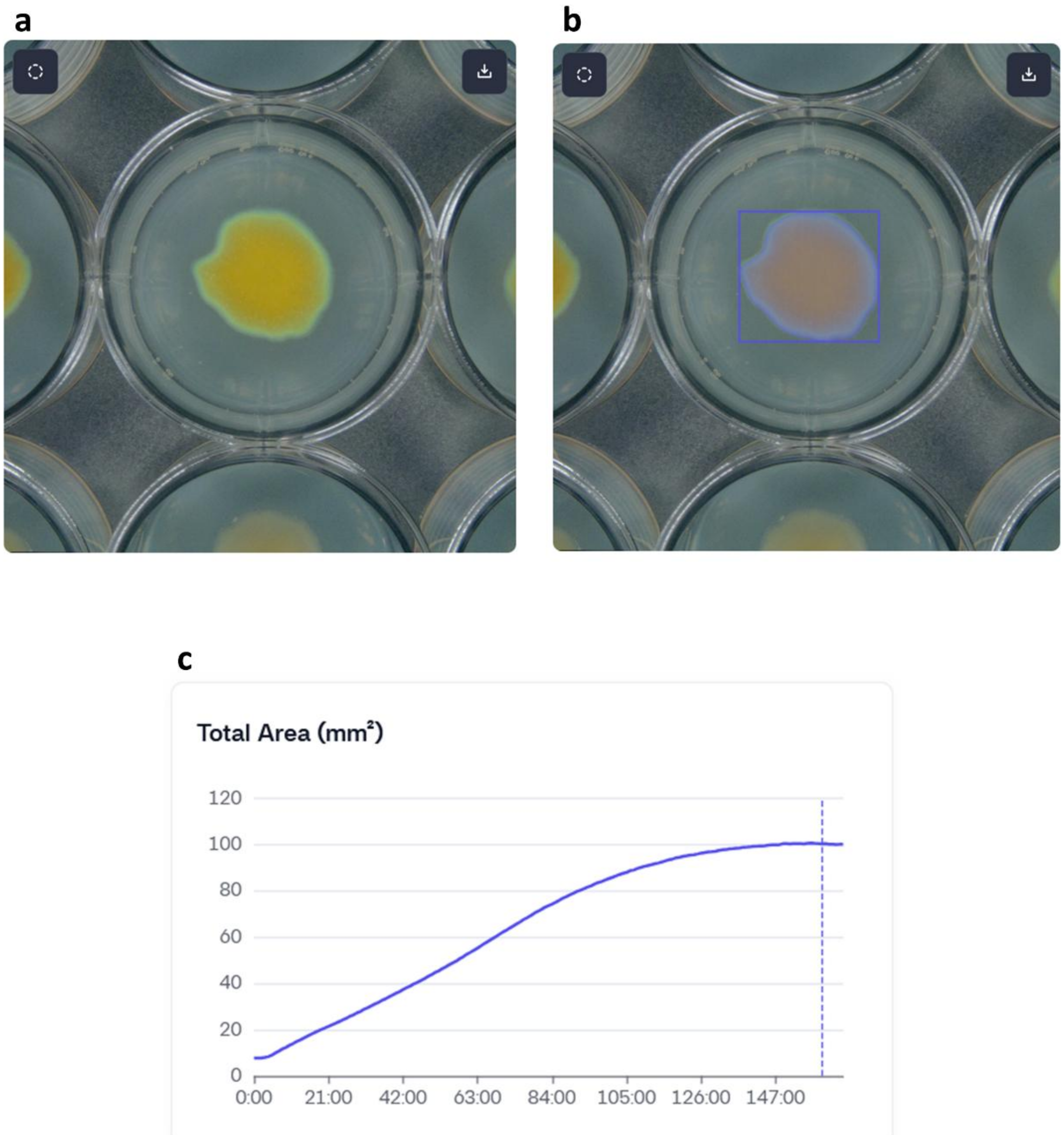

Sup. fig. 1: Automated annotation and measurement of colony areas *via* Reshape Biotech imaging platform. Macro-colonies were cultivated on marine agar and imaged over time using the Reshape Biotech automated system. **a**, Shows a raw colony image, while **b**, displays the corresponding annotated image generated by the platform's integrated deep learning algorithm. **c**, The colony area was measured at hourly intervals, represented by the resulting area-over-time curve.

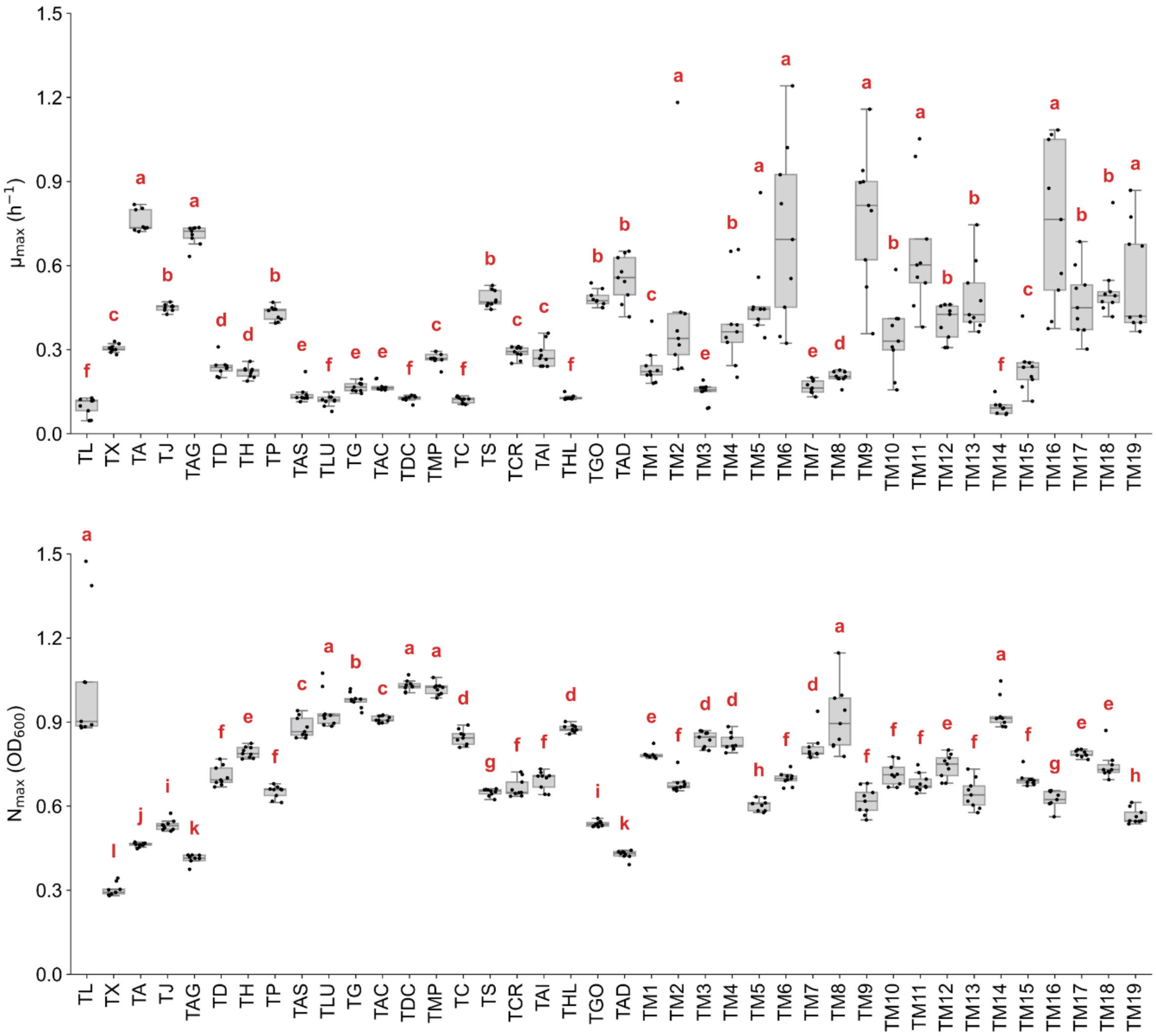

Sup. fig. 2: Planktonic model: distribution of maximum specific growth rates (μ_{max}) and maximum population density (N_{max}) across *Tenacibaculum* strains. Each boxplot shows the distribution of biological and technical replicates for individual strains. Letters (a, b, c, etc.) represent greedy disjoint similarity groups (Games-Howell, $p > 0.05$). Shared letters indicate statistical similarity among grouped strains. Differing letters do not necessarily denote significant differences among strains across groups.

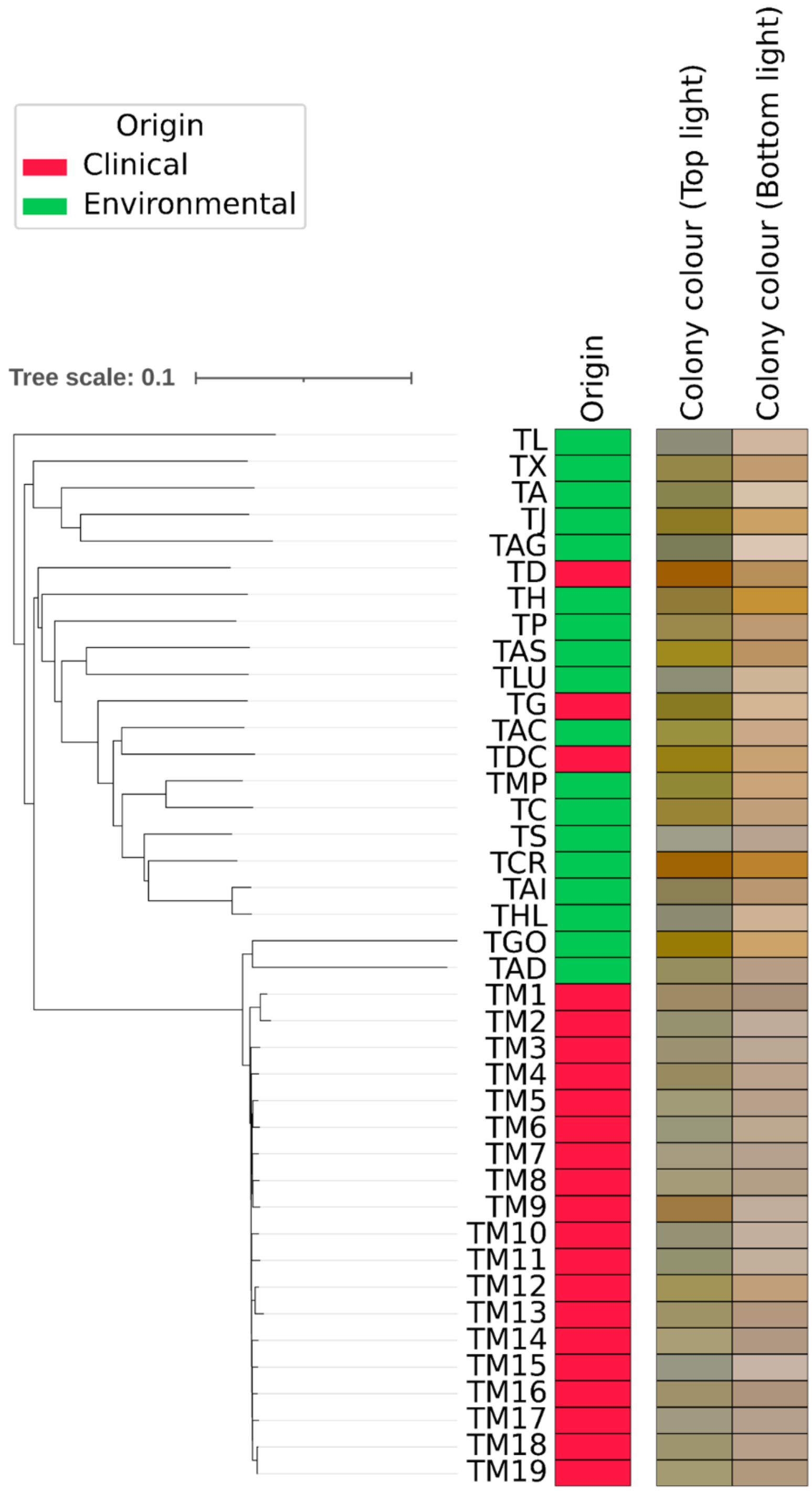

Sup. fig. 3: Image-based quantification of macro-colony colouration in clinical and environmental *Tenacibaculum* isolates. Mean spectral signatures (RGB) were derived from ≥5 biological replicates per isolate.

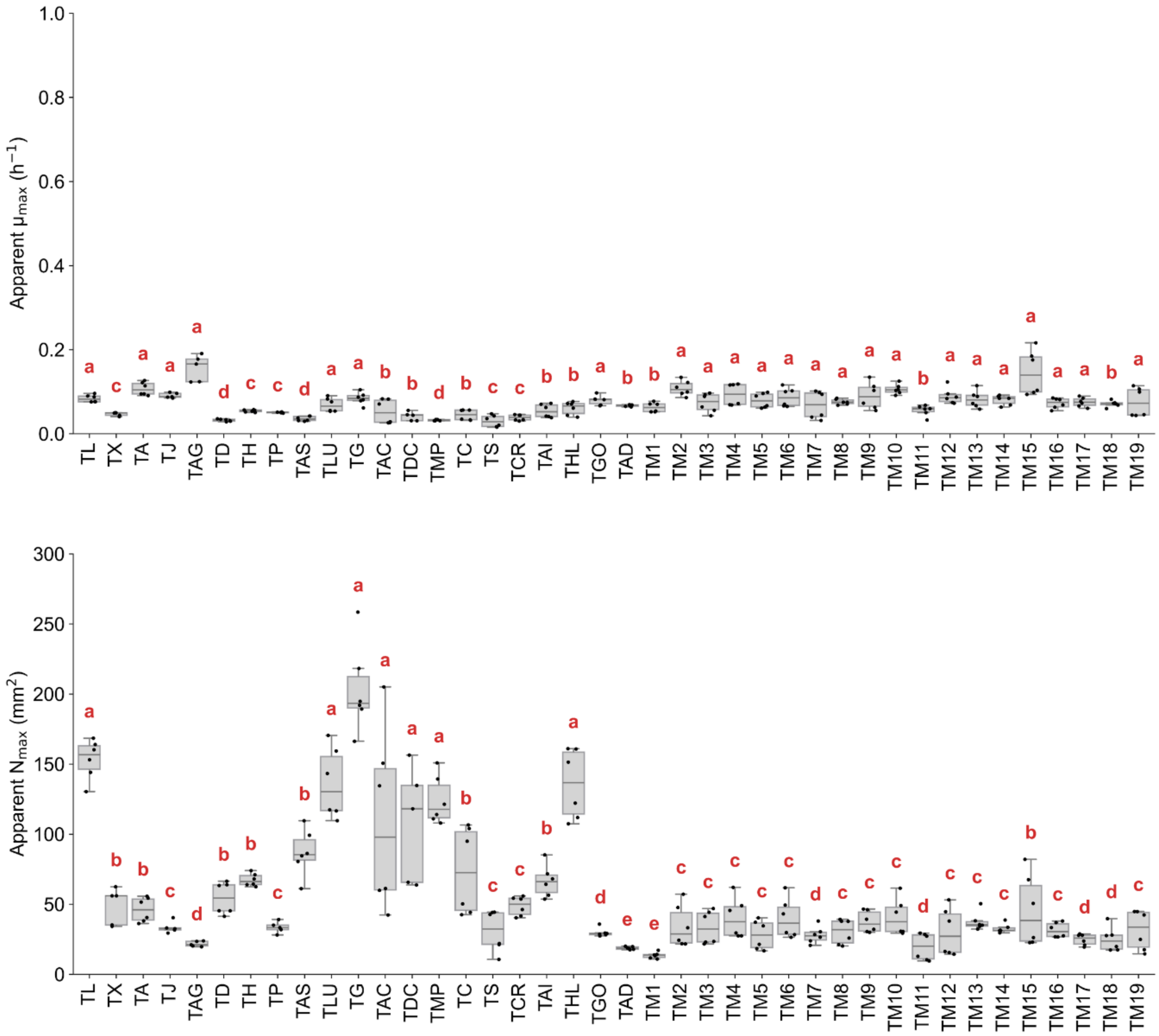

Sup. fig. 4: Macro-colony model: distribution of apparent maximum growth rate (apparent μ_{max}) and apparent maximum colony size (apparent N_{max}) across *Tenacibaculum* strains. Each boxplot shows the distribution of biological and technical replicates for individual strains. Letters (a, b, c, etc.) represent greedy disjoint similarity groups (Games-Howell, $p > 0.05$). Shared letters indicate statistical similarity among grouped strains. Differing letters do not necessarily denote significant differences among strains across groups.

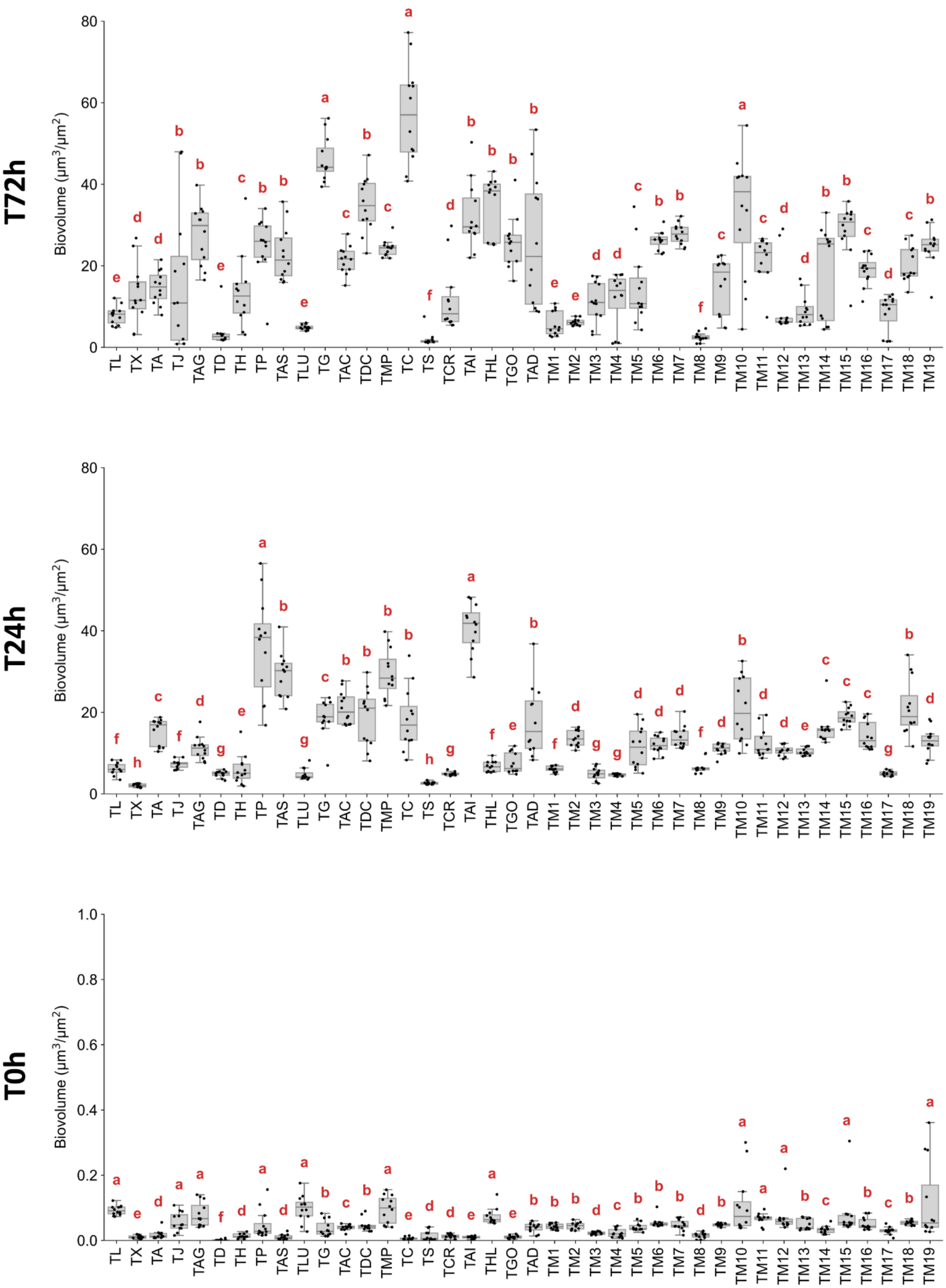
T72h
T24h
T0h
Biovolume (µm³/µm²)
TL TX TA TJ TAG TD TH TP TAS TLU TG TAC TDC TMP TC TS TCR TAI THL TGO TAD TM1 TM2 TM3 TM4 TM5 TM6 TM7 TM8 TM9 TM10 TM11 TM12 TM13 TM14 TM15 TM16 TM17 TM18 TM19

Sup. fig. 5: Submerged biofilm model: quantification of biofilm biovolume across *Tenacibaculum* strains at three time points (T0h, T24h & T72h). Each boxplot shows the distribution of biovolume values across biological and technical replicates for individual strains. Letters (a, b, c, etc.) represent greedy disjoint similarity groups (Games-Howell, $p > 0.05$). Shared letters indicate statistical similarity among grouped strains. Differing letters do not necessarily denote significant differences among strains across groups.

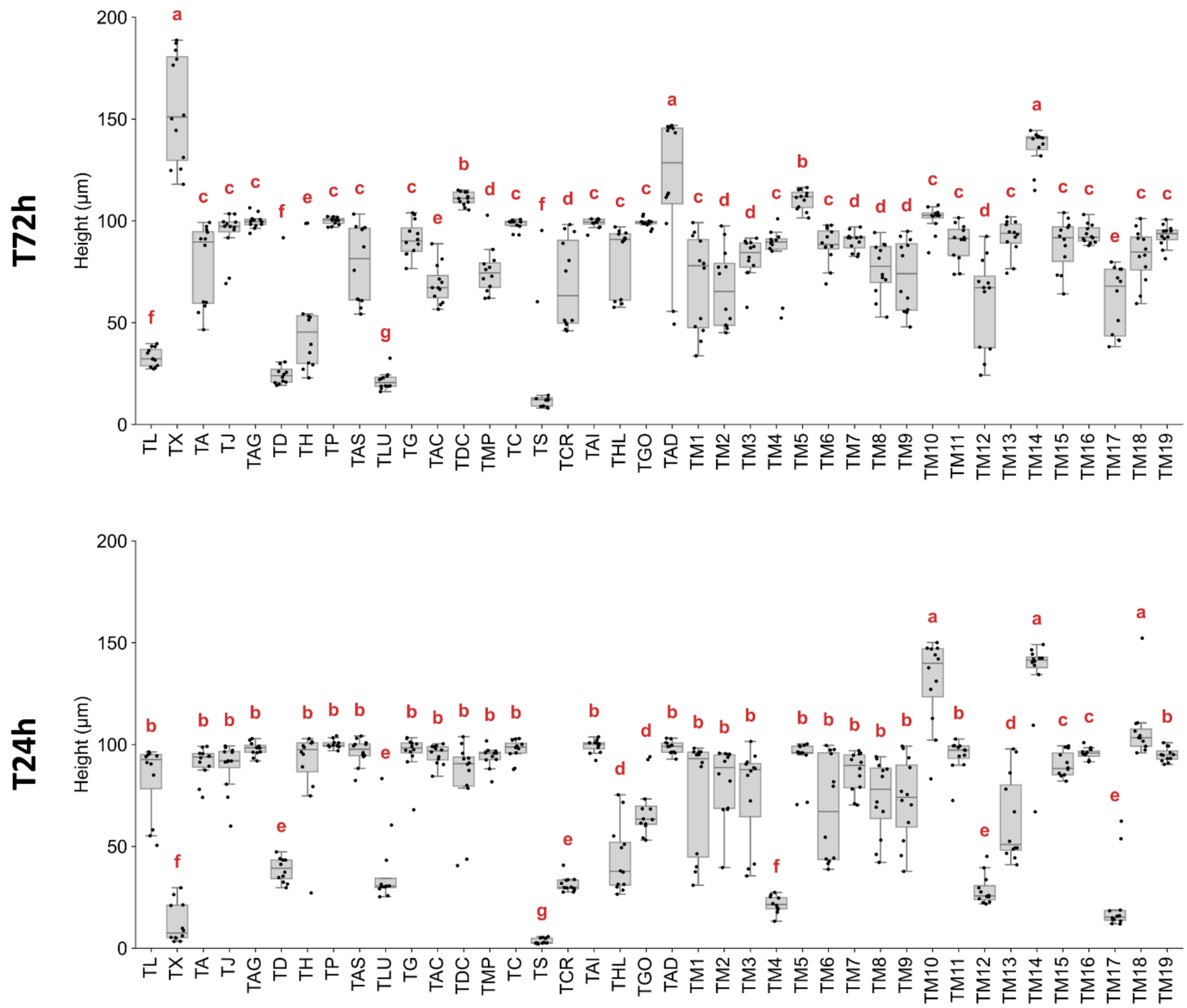

Sup. fig. 6: Submerged biofilm model: quantification of biofilm height across *Tenacibaculum* strains at two time points (T24h & T72h). Each boxplot shows the distribution of biovolume values across biological and technical replicates for individual strains. Letters (a, b, c, etc.) represent greedy disjoint similarity groups (Games-Howell, $p > 0.05$). Shared letters indicate statistical similarity among grouped strains. Differing letters do not necessarily denote significant differences among strains across groups.

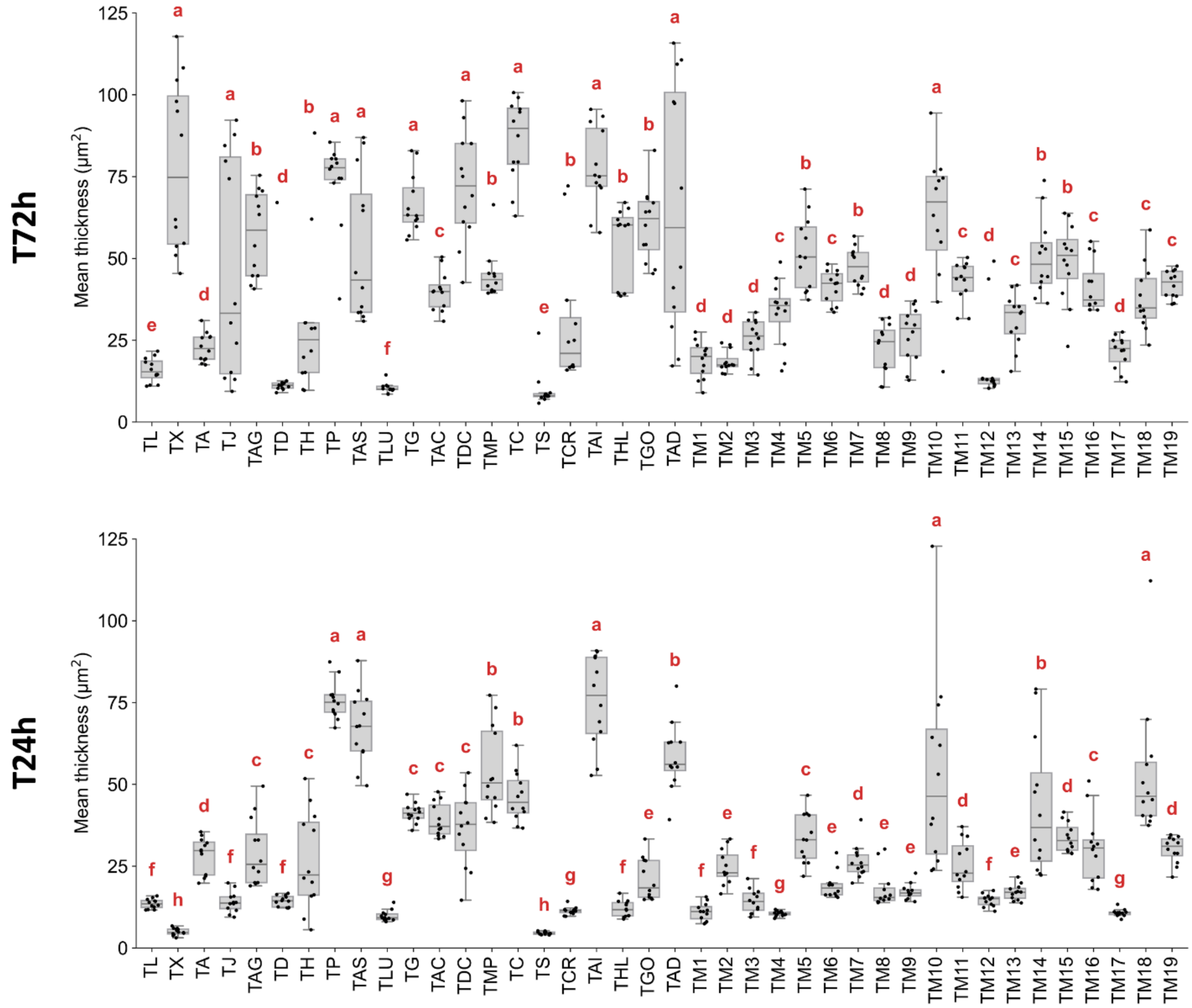

Sup. fig. 7: Submerged biofilm model: quantification of biofilm mean thickness across *Tenacibaculum* strains at two time points (T24h & T72h). Each boxplot shows the distribution of biovolume values across biological and technical replicates for individual strains. Letters (a, b, c, etc.) represent greedy disjoint similarity groups (Games-Howell, $p > 0.05$). Shared letters indicate statistical similarity among grouped strains. Differing letters do not necessarily denote significant differences among strains across groups.

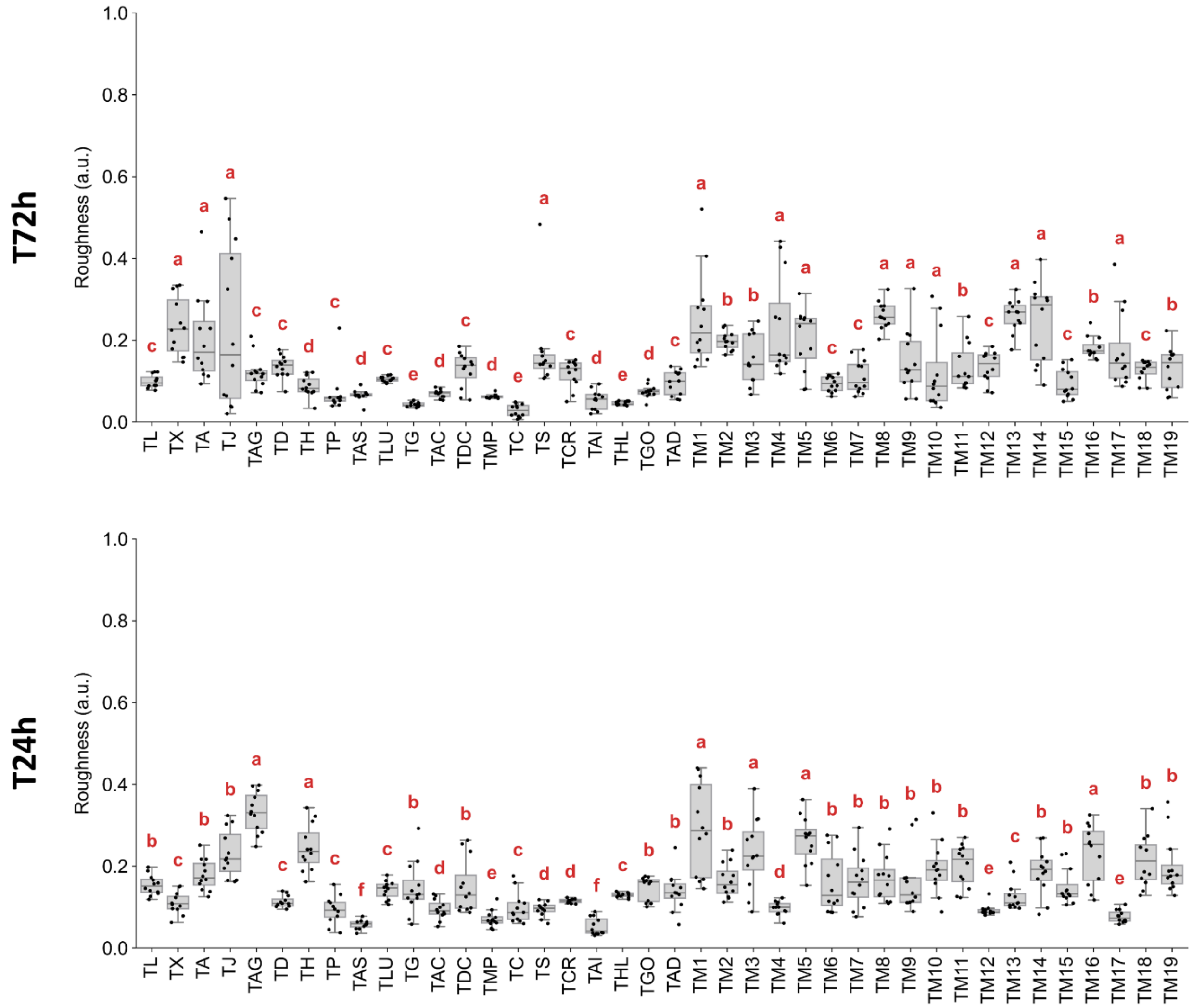

Sup. fig. 8: Submerged biofilm model: quantification of biofilm roughness across *Tenacibaculum* strains at two time points (T24h & T72h). Each boxplot shows the distribution of biovolume values across biological and technical replicates for individual strains. Letters (a, b, c, etc.) represent greedy disjoint similarity groups (Games-Howell, $p > 0.05$). Shared letters indicate statistical similarity among grouped strains. Differing letters do not necessarily denote significant differences among strains across groups.

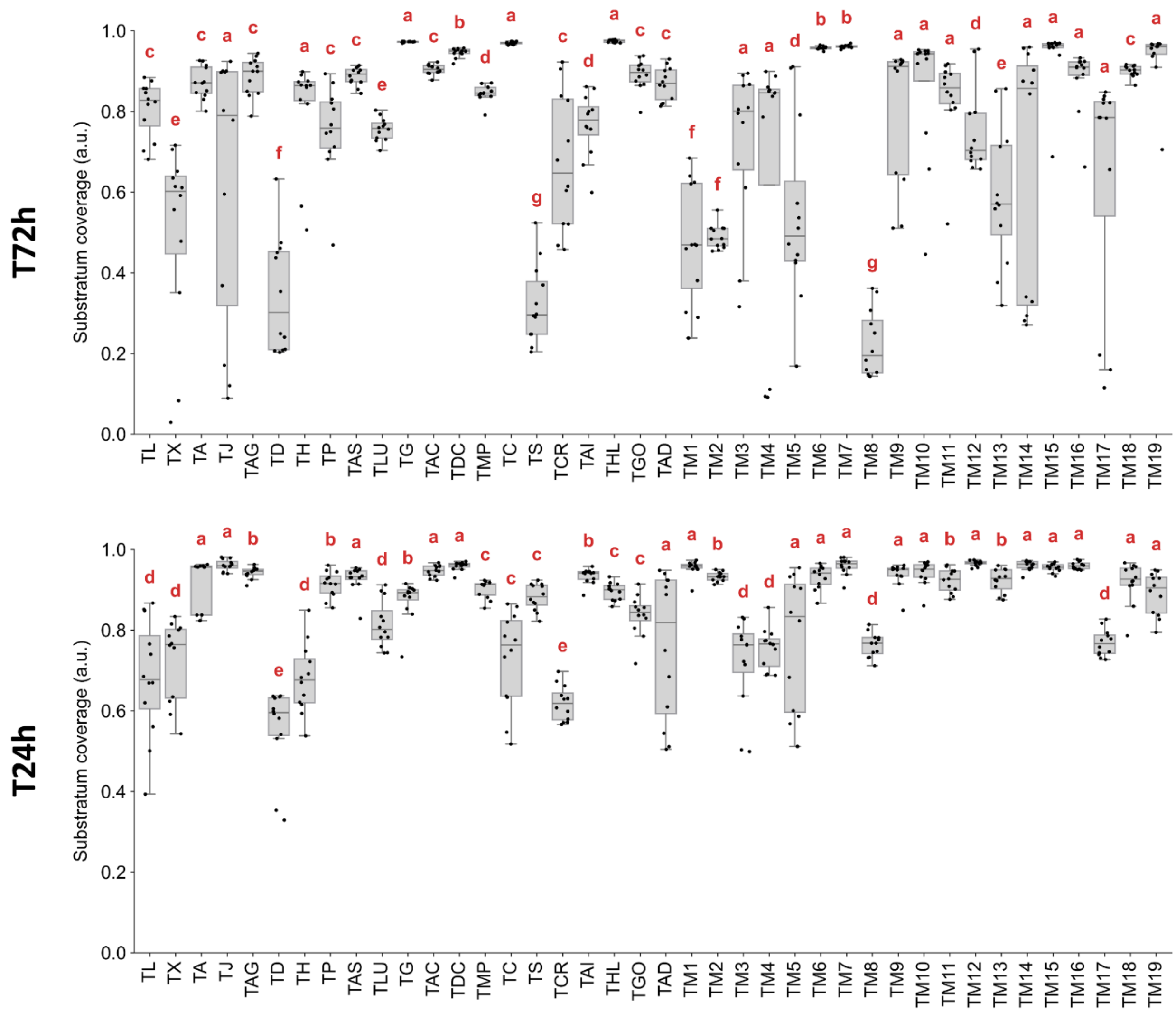

Sup. fig. 9: Submerged biofilm model: quantification of biofilm substratum coverage across *Tenacibaculum* strains at two time points (T24h & T72h). Each boxplot shows the distribution of biovolume values across biological and technical replicates for individual strains. Letters (a, b, c, etc.) represent greedy disjoint similarity groups (Games-Howell, $p > 0.05$). Shared letters indicate statistical similarity among grouped strains. Differing letters do not necessarily denote significant differences among strains across groups.

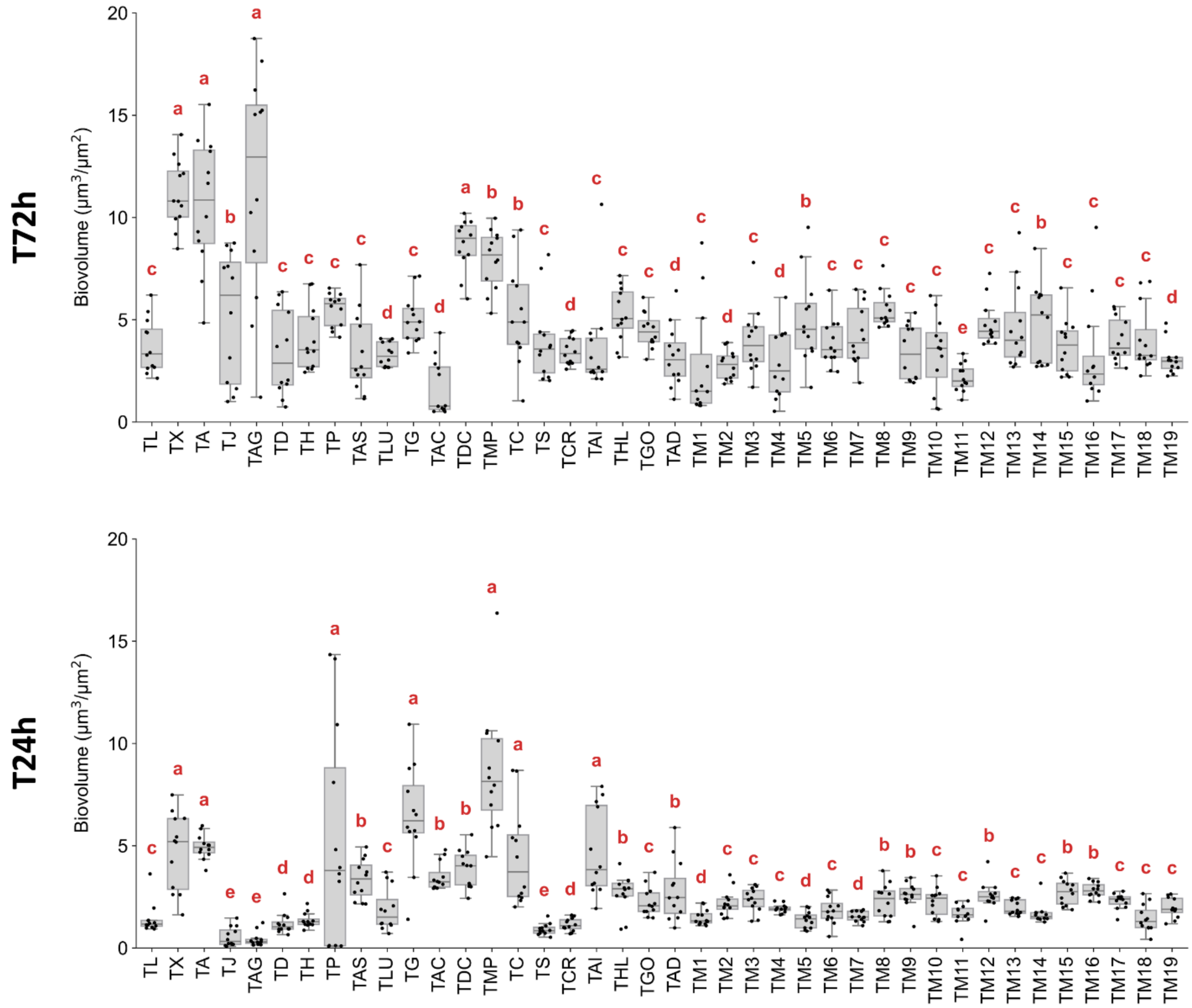

Sup. fig. 10: Submerged biofilm model: quantification of Congo red labelled matrix (CR-matrix) biovolume across *Tenacibaculum* strains at two time points (T24h & T72h). Each boxplot shows the distribution of CR-matrix biovolume values across biological and technical replicates for individual strains. Letters (a, b, c, etc.) represent greedy disjoint similarity groups (Games-Howell, $p > 0.05$). Shared letters indicate statistical similarity among grouped strains. Differing letters do not necessarily denote significant differences among strains across groups.

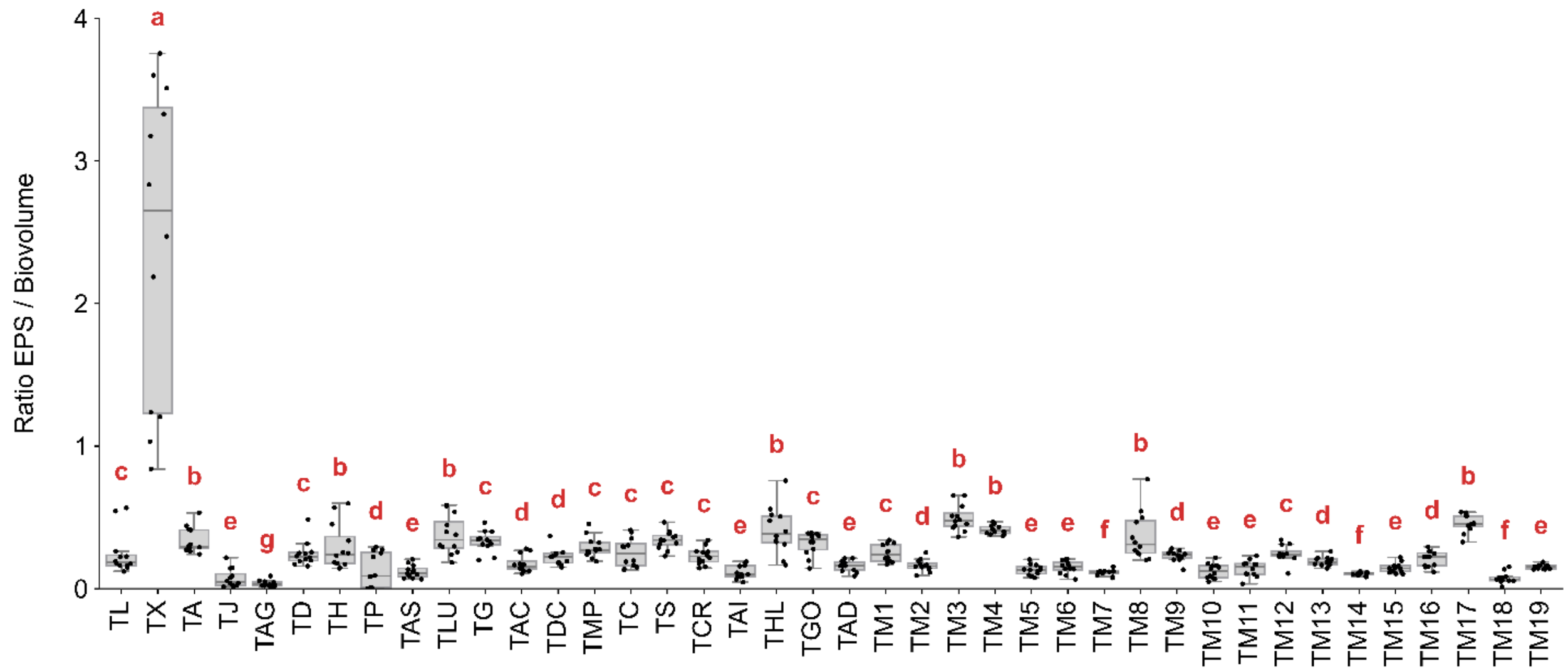

Sup. fig. 11: Submerged biofilm model: Congo red labelled matrix biovolume-to-bacterial biomass ratios across *Tenacibaculum* strains at 24 hours. Each boxplot shows the distribution of matrix-to-bacterial biovolume ratios across biological and technical replicates for individual strains. Letters (a, b, c, etc.) represent greedy disjoint similarity groups (Games-Howell, $p > 0.05$). Shared letters indicate statistical similarity among grouped strains. Differing letters do not necessarily denote significant differences among strains across groups.

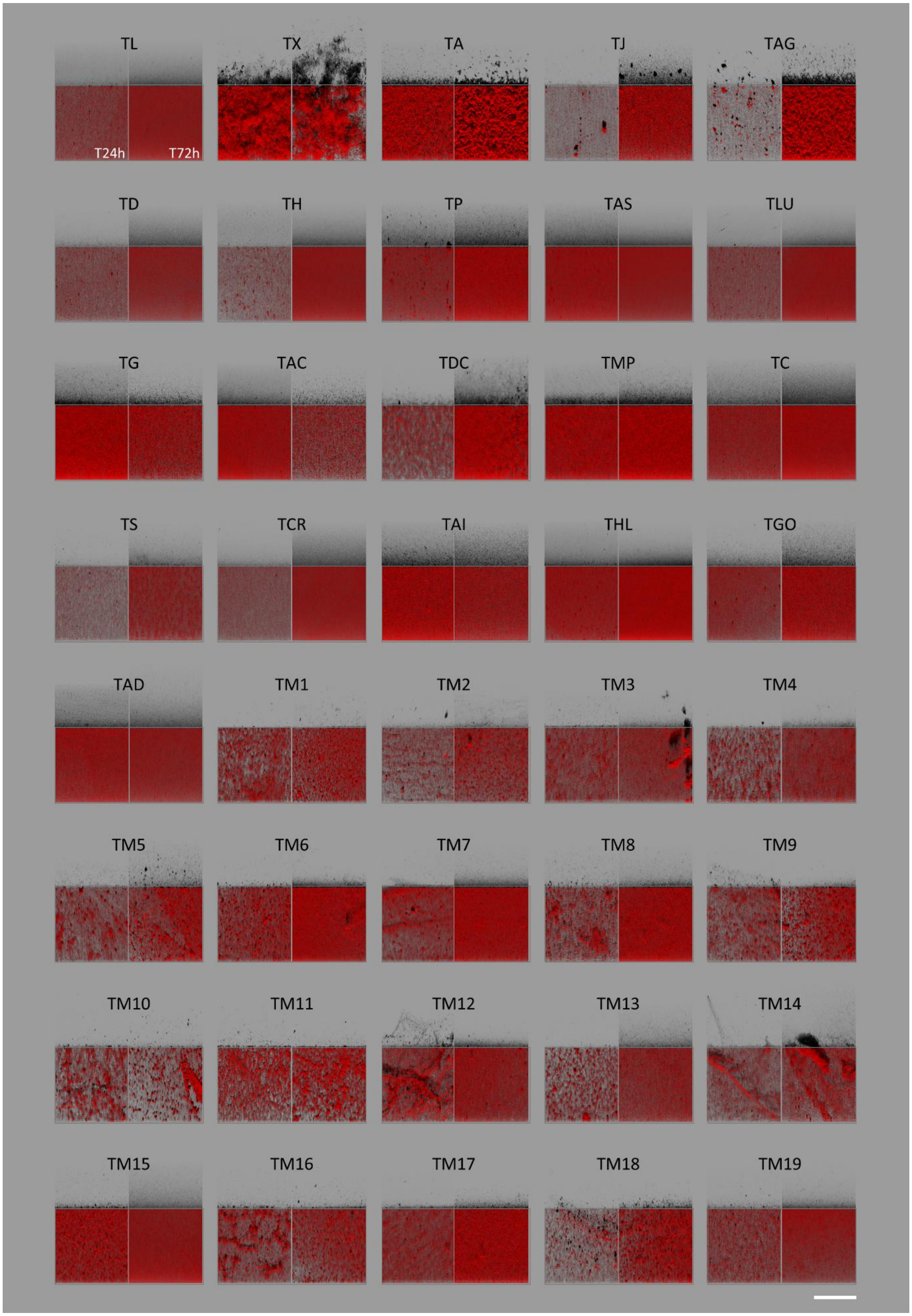
TL
TX
TA
TJ
TAG
T24h
T72h
TD
TH
TP
TAS
TLU
TG
TAC
TDC
TMP
TC
TS
TCR
TAI
THL
TGO
TAD
TM1
TM2
TM3
TM4
TM5
TM6
TM7
TM8
TM9
TM10
TM11
TM12
TM13
TM14
TM15
TM16
TM17
TM18
TM19

Sup. fig. 12: Architectural diversity of the Congo red labelled *Tenacibaculum* spp. matrix material. Representative images illustrate the spatial distribution and biovolume of the putative matrix material across 40 *Tenacibaculum* strains. Snapshots were acquired at two developmental stages: T24h (maturation) and T72h (late-stage development). The biofilm matrix is visualised *via* Congo Red labelling (red), highlighting amyloid fibre production and structural components of the self-produced matrix. The 2D projections were generated from confocal z-stacks using IMARIS software in blend mode. Scale bar = 40µm.

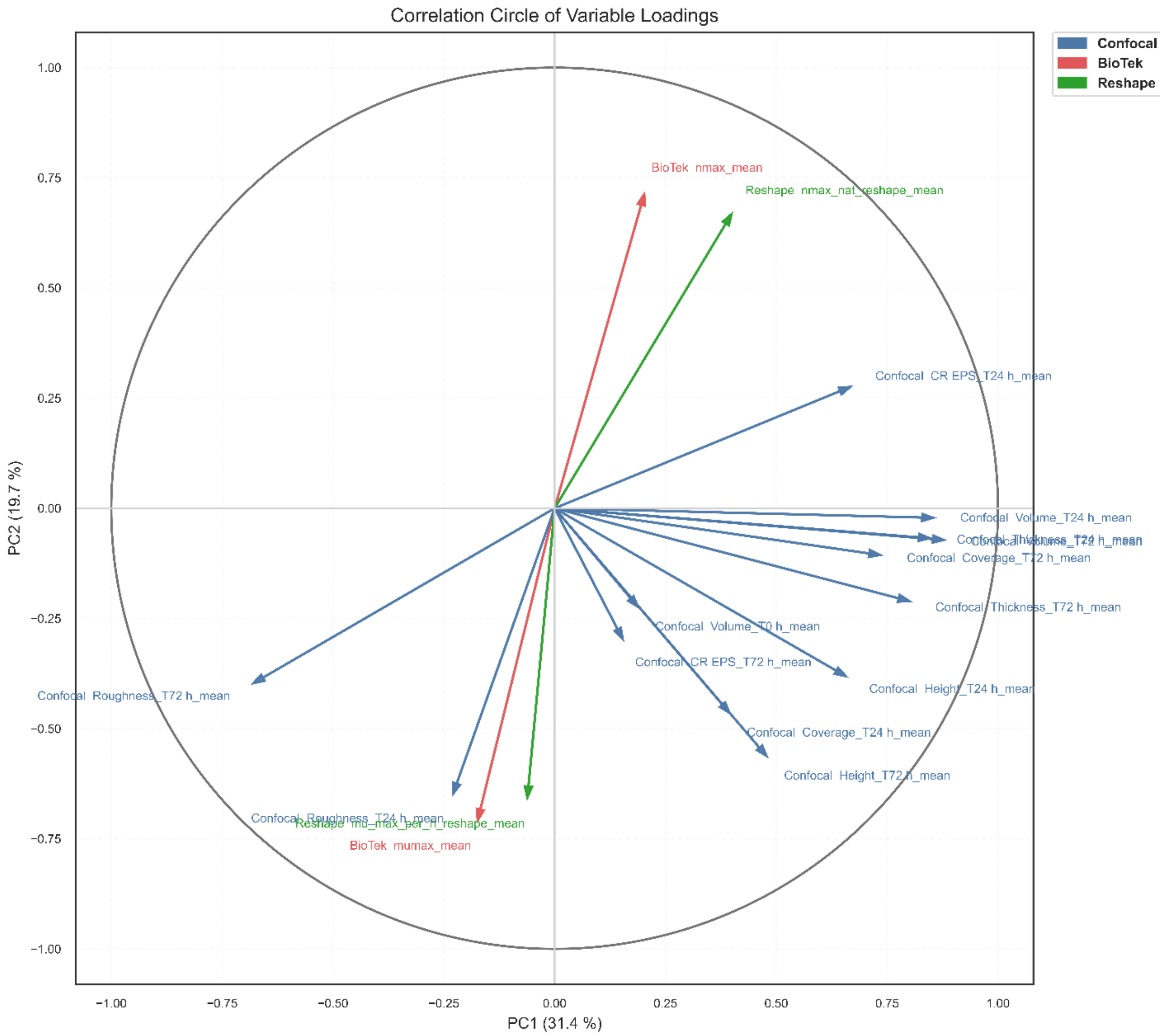

Sup. fig. 13: Correlation circle of variable loadings for the first two principal components. Principal component analysis (PCA) was performed on 17 normalised phenotypic variables comprising planktonic growth, macro-colony expansion, and submerged biofilm architecture. PC1 and PC2 explain 31.4% and 19.7% of the total variance, respectively. Vector direction and length indicate the strength and contribution of each variable to the corresponding principal component.

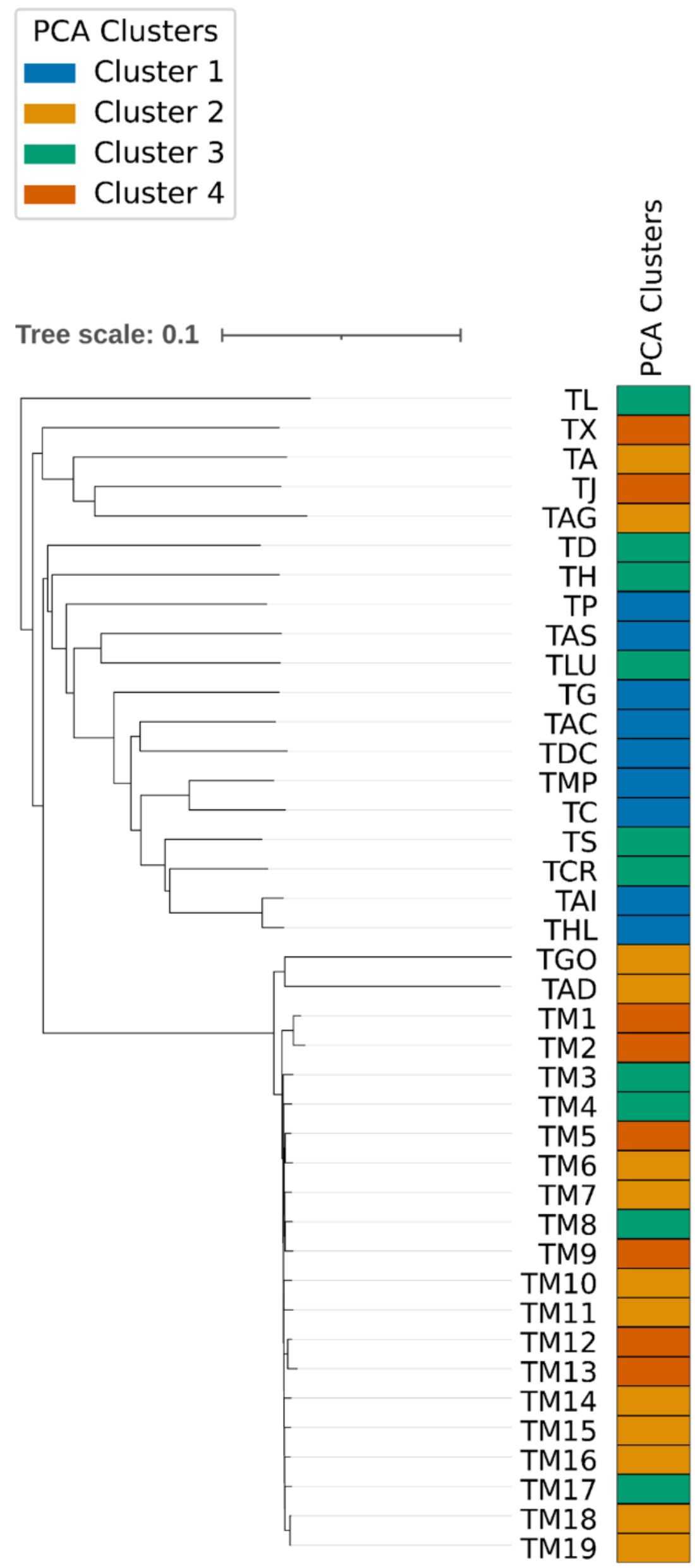

Sup. fig. 14: Distribution of phenotypic clusters across the *Tenacibaculum* collection. A neighbour-joining phylogenetic tree of 40 *Tenacibaculum* isolates was constructed from a Mash distance matrix, with branch lengths representing 1–ANI (Average Nucleotide Identity). Phenotypic clusters identified via multivariate analysis were mapped alongside the tree as a colour-coded metadata strip to visualise the relationship between genetic similarity and developmental strategies.

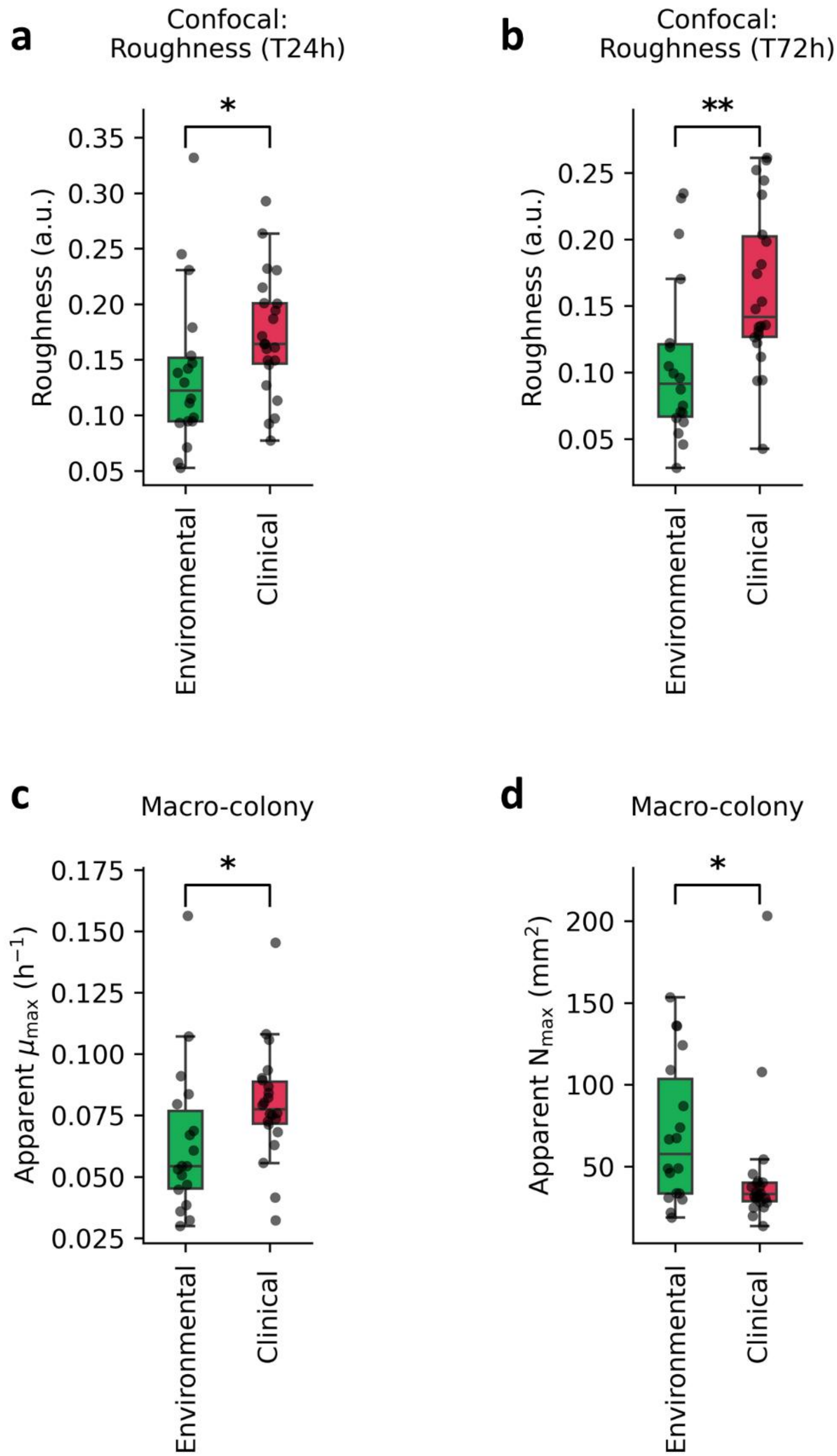

Sup. fig. 15: Phenotypic parameters highlighting significant divergence between environmental and clinical *Tenacibaculum* isolates. Panels **a–b**, present two parameters from the submerged

biofilm model that showed significant differences based on the origin of the isolate; clinical strains exhibited higher biofilm roughness at both T24h and T72h compared to environmental isolates. Parameters derived from the macro-colony biofilm model, shown in panels **c–d**, revealed that clinical strains reached a higher apparent maximum growth rate (apparent μ_{max}), whereas environmental isolates produced a significantly larger apparent maximum colony size (apparent N_{max}).

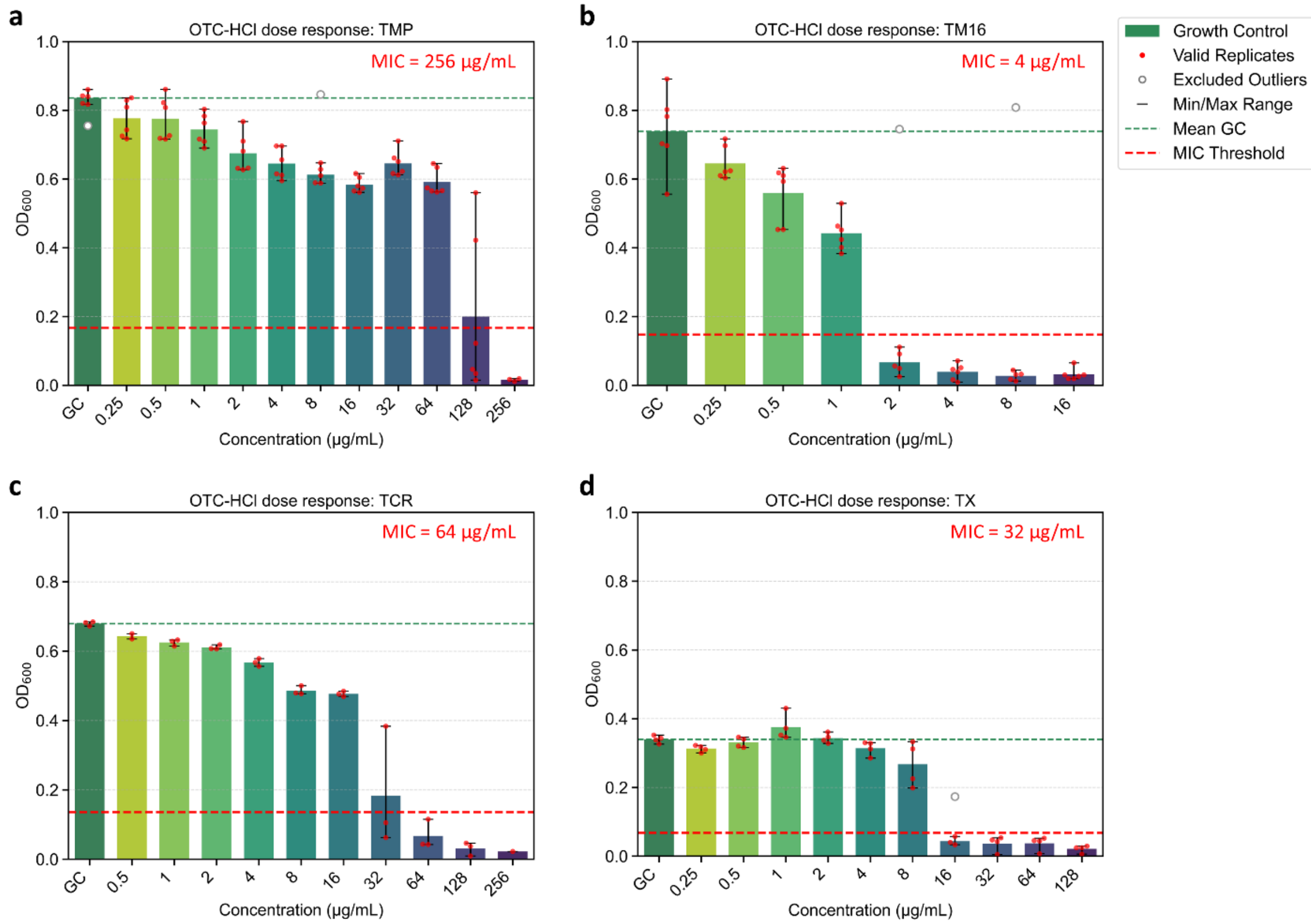

Sup. fig. 16: Dose-response profiles and determination of minimum inhibitory concentration (MIC) of Oxytetracycline hydrochloride (OTC-HCl) against representative *Tenacibaculum* strains. **a**, For strain TMP, the MIC was determined to be 256 µg/mL. **b**, TM16 demonstrated a relatively lower MIC of 4 µg/mL. **c**, The growth of TCR reached the inhibition threshold at 64 µg/mL. **d**, An MIC of 32 µg/mL was recorded for strain TX. For each panel, the dashed red line signifies the 80% inhibition threshold calculated relative to that specific strain's mean growth control (green dashed line).

Supplementary Video

Sup. video 1: Time-lapse monitoring of *Tenacibaculum* macro-colony development. The video illustrates the growth and expansion kinetics of 40 *Tenacibaculum* strains over a week at 28°C on marine agar. Labels correspond to the primary strain IDs representing the collection of *Tenacibaculum* isolates. Unlabelled wells represent samples excluded from the final analysis. For every strain, 2-3 biological replicates were observed.
